# Ultra-highly linear Ga_2_O_3_-based cascade heterojunctions optoelectronic synapse with thousands of conductance states for neuromorphic visual system

**DOI:** 10.1038/s41377-025-01897-9

**Published:** 2025-09-30

**Authors:** Peng Li, Xuanyu Shan, Ya Lin, Yi Du, Jiangang Ma, Zhongqiang Wang, Xiaoning Zhao, Ye Tao, Haiyang Xu, Yichun Liu

**Affiliations:** Key Laboratory for UV Light-Emitting Materials and Technology of Ministry of Education, Northeast Normal University, Changchun, China

**Keywords:** Photonic devices, Nonlinear optics

## Abstract

Ultrawide bandgap semiconductor optoelectronic synapses can perform high-parallel computing with a low false alarm rate, making them ideal for building deep-ultraviolet (DUV) neuromorphic visual system (NVS). However, the rapid carrier recombination in these optoelectronic synapses results in a poor number of conductance states and a low linear weight update protocol, consequently degrading the image recognition accuracy of DUV NVSs. This work proposes a type of cascade heterojunctions capable of finely tuning the dynamics of photogenerated carriers, utilizing aluminum interdigital electrodes sandwiched between tin-doped Ga_2_O_3_ and oxygen-deficient hafnium oxide (GTO/Al/HfO_x_) films. The built-in fields at the GTO/HfO_x_ heterojunction and the Al/HfO_x_ hole Schottky junction interfaces facilitate the separation of photogenerated carriers and the subsequent trapping of holes by the oxygen defects in the HfO_x_, respectively. The GTO/Al/HfO_x_ optoelectronic synapses exhibit an ultrahigh responsivity of over 10^4^ A/W and a large photo-to-dark current ratio of 6 × 10^5^, which results in exceptional synaptic plasticity with unprecedented 4096 conductance states and excellent linearity with a fitting coefficient of 0.992. These attributes enable the GTO/Al/HfO_x_ optoelectronic synapses to execute logical operations with fault-tolerance capability and to achieve high-accuracy fingerprint classification. The innovative cascade heterojunctions design, along with the elucidated carrier dynamics modulation mechanism, facilitates the development of DUV NVSs.

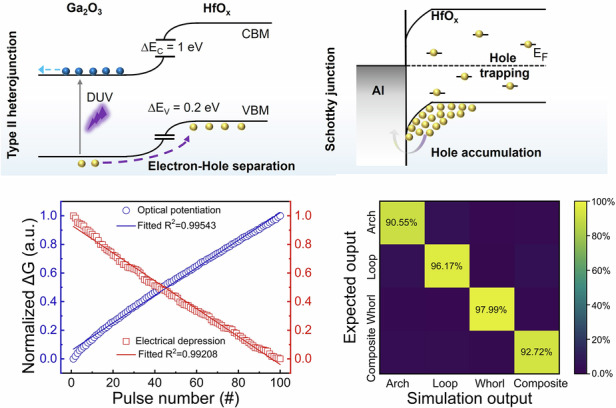

## Abstract (web summary)

The Ga_2_O_3_:Sn/Al/HfO_x_ cascade heterojunctions-based optoelectronic synapses exhibit outstanding linearity in weight updating and thousands of conduction levels, holding promise for applications in logic operations, arithmetic computing, and fingerprint classification.

## Introduction

Biologies acquire external visual information through a collaborative cooperation between the eyes and the brain. The retina detects and preprocesses this visual data and then relays it to the visual cortex for more sophisticated perceptual analysis. Neuromorphic visual systems (NVS) draw inspiration from the biological visual system, replicating its in-sensor computing capabilities with a fusion of information sensing, memory, and processing. Unlike traditional computer vision systems, which are based on von Neumann architectures and are hindered by decision-making delays, the all-in-one NVS offers a highly compact solution that streamlines circuit design, enhances processing efficiency, and diminishes power consumption. In the fast-evolving landscape of electronic technologies, including artificial intelligence and the Internet of Things, the development of NVS is attracting growing interest and is emerging as a leading edge in research^1,2^.

Deep ultraviolet (DUV) radiation, an invisible component of solar radiation that is largely absorbed by the upper atmosphere and does not reach the Earth’s surface, exhibits minimal noise interference. This characteristic makes DUV information perception and processing critical for a wide range of vital uses in military and economic sectors, such as missile warning, environmental monitoring, and secure communications etc. Additionally, the intense absorption by organic residues confers DUV light with exceptional potential for the identification and classification of fingerprints. To meet these applications, DUV NVSs emerge as nascent, revolutionary technologies in recent years. While visible, UV, and infrared NVSs have seen recent implementation^3–5^, the advancement of DUV NVSs has lagged behind and their image recognization accuracy is limited by the scarcity of DUV optoelectronic synapses with linearly programmable plasticity and multiple conductance levels. Conventional complementary metal-oxide-semiconductor (CMOS)-based optoelectronic synapses, composed of narrow bandgap semiconductors, are mature and compatible with current semiconductor technologies. However, their broad spectral response and weak DUV sensitivity limit their suitability for DUV NVSs. Similarly, other semiconductors, such as two-dimensional materials, perovskites, and traditional wide-bandgap materials like InGaZnO, suffer from low DUV-to-visible suppression ratios, making them better suited for longer wavelengths^5–7^. In contrast, ultrawide bandgap semiconductors (e.g., diamond, AlGaN, and Ga_2_O_3_) with bandgaps >4.5 eV are ideal for DUV NVSs due to their inherent DUV selectivity and strong anti-visible interference capability. Among these, Ga_2_O_3_ excels due to its processability, tunable electrical properties, high DUV absorption coefficient, and robust stability^8^. Ga_2_O_3_-based synapses also exhibit high resistivity and superior power efficiency compared to CMOS-based technologies. Moreover, their fabrication via low-cost, scalable physical or chemical vapor deposition methods ensures compatibility with existing integration processes, facilitating seamless adoption in semiconductor platforms. Recent pioneering research has developed Ga_2_O_3_-based optoelectronic synapses using metal/semiconductor/metal structure^9,10^. Nevertheless, the rapid electron-hole recombination process limits the overall performance of these optoelectronic synapses.

Manipulating carrier dynamics, including separation, transport, and collection, is pivotal in controlling the responsivity and response time of optoelectronic synapses, potentially offering solutions for highly efficient DUV NVSs. Traditional strategies, such as creating heterojunction^11–13^ and Schottky junction^14^ and the incorporation of pyroelectric‒ or acousto‒photoelectric coupling effects^15–17^ can enhance the responsivity of DUV optoelectronic synapses. However, the duration of the linear current increase/decrease during DUV illumination in the optoelectronic synapses was unavoidably shortened, which could lead to a reduction in the number of accessible conductance states. An alternative strategy involves the construction of multiple heterojunctions, which could promote carrier separation and subsequent defect-assisted trapping of minority carriers without influencing the transport of majority carriers, thereby achieving high gain to boost responsivity and to extend the linear increase/decrease duration of the photocurrent. However, the matching of ultra-wide bandgap semiconductor and metal electrode materials with appropriate energy band alignments, as well as the design of feasible device structures to facilitate these physical processes, remains an ongoing challenge.

Hafnium oxide (HfO_x_), a transition metal oxide compatible with CMOS technology, has been thoroughly investigated as a high-k dielectric for semiconductor manufacturing and shows promise for advancing microelectronic devices such as ferroelectric and resistive random-access memories. An attribute of transition metal oxides is that their electronic characteristics are profoundly affected by stoichiometry and defect chemistry, which allows HfO_x_ films with substoichiometric compositions to exhibit semiconducting properties because the oxygen vacancy defect band that appears below the Fermi level and above the valence band maximum (VBM) in HfO_x_^18–20^. Further harnessing the high conduction band minimum (CBM), which originates from the 5 d orbitals of hafnium, oxygen-deficient HfO_x_ is positioned as a prime candidate for forming heterojunction with Ga_2_O_3_, thereby promoting the separation of electron-hole pairs and the subsequent trapping of holes to induce gain.

**Fig. 1:**
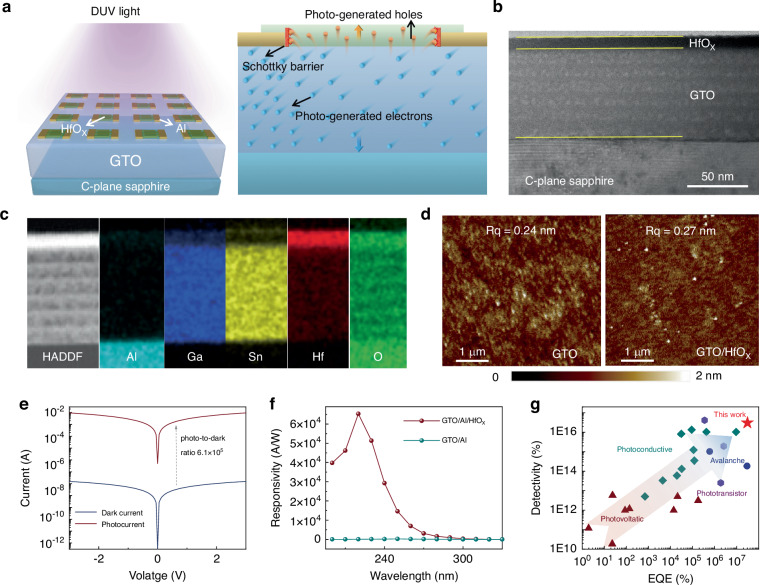
Design of GTO/Al/HfO_x_ cascade heterojunctions. **a** Illustration depicting the planar and cross-sectional architecture of the cascade heterojunctions, featuring Al interdigitated electrodes sandwiched between the GTO and HfO_x_ layers. Upon DUV light exposure, photogenerated electron-hole pairs are separated at the GTO/HfO_x_ interface. Electrons traverse the GTO layer and are collected by the Al electrode, while holes are impeded by the Schottky barrier at the Al/HfO_x_ interface and become trapped in oxygen defects within HfO_x_. **b** Cross-sectional HAADF-STEM image of the GTO/HfO_x_ heterojunction on the sapphire substrate, and **c** corresponding energy dispersive spectrum mapping for Al, Ga, Sn, Hf, and O elements. **d** AFM images of the GTO and GTO/HfO_x_ heterojunction on the sapphire substrate. **e** I-V characteristics of the GTO/Al/HfO_x_ cascade heterojunctions without and with DUV light exposure. **f** Responsivity as a function of light wavelength for the GTO/Al device and GTO/Al/HfO_x_ cascade heterojunctions. **g** Performance benchmark, highlighting the superior detectivity at substantial EQE when compared to Ga_2_O_3_-based DUV photoconductive sensors^21^, photovoltaic sensors^14,15^, phototransistors, and avalanche diodes

This work develops GTO/Al/HfO_x_ cascade heterojunctions featuring aluminum interdigitated electrodes sandwiched between tin-doped Ga_2_O_3_ (GTO) and oxygen-deficient HfO_x_ films (Fig. 1a). The involved GTO/HfO_x_ type-II heterojunction facilitates the separation of photogenerated electron-hole pairs, and the Al/HfO_x_ hole Schottky barrier prevents hole collection by the Al electrode, instead promoting their trapping by oxygen vacancies within the HfO_x_ layer. This hole-trapping-induced gain endows the cascade heterojunctions optoelectronic synapses with an ultrahigh DUV responsivity of over 10^4^ A/W, unprecedented conductance states of 4096, and linearity with a fitting coefficient of 0.992. The GTO/Al/HfO_x_ NVSs can perform logical operations with fault-tolerance capability and achieve high-accuracy fingerprint classification, representing a significant advancement of neuromorphic computing techniques toward DUV applications.

## Results

### Design and photoresponse of GTO/Al/HfO_x_ cascade heterojunctions

Figure 1a depicts the schematic of a 4 × 4 GTO/Al/HfO_x_ cascade heterojunctions array, along with a cross-sectional illustration of an individual device. The microstructure and elemental composition of the GTO/HfO_x_ heterostructure were investigated by high-angle annular dark-field scanning transmission electron microscopy (HAADF-STEM) and energy dispersive spectroscopy (EDS) analyses, as shown in Fig. 1b and c. The HfO_x_ film, fabricated through magnetron sputtering in an oxygen-free environment, is characterized by its compactness, and the GTO film, prepared by a process of repeated spin-coating followed by annealing, exhibits a multilayered stack. The EDS analyses illustrate the distribution of Al, Sn, Ga, O, and Hf, with the Al signal originating from the sapphire substrate. The GTO and HfO_x_ films are amorphous with low root-mean-square roughness of 0.24 nm and 0.27 nm, respectively (Fig. 1d and Supplementary Fig. S1). These low-roughness surfaces of GTO and GTO/HfO_x_ provide the foundation for achieving heterojunctions with high optoelectronic performance.

Figure 1e illustrates the semilogarithmic current-voltage characteristics of the GTO/Al/HfO_x_ cascade heterojunctions, measured in both dark conditions and under 254-nm illumination, with an applied bias ranging from ‒3 to 3 V. The device exhibited a high on-off current ratio of 6.1 × 10^5^. The maximum responsivity (*R*) of the GTO/Al/HfO_x_ cascade heterojunctions, calculated using the equation *R* = (*I*_illumination_–*I*_dark_)/(*PS*) = *I*_P_/(*PS*), where *I*_P_ denotes the photocurrent of 9.4 × 10^−3^ A, *P* represents the light power density of 21 μW/cm^2^, and *S* is the surface area of 7 × 10^−3^ cm^2^, reaches 6.5 × 10^4^ A/W (Fig. 1f). This value is approximately 300-fold greater than that of similar structures lacking the HfO_x_ layer. Supplementary Table S1 provides a detailed performance comparison of the other control devices that were developed throughout the optimization process. It is noteworthy that the peak responsivity of GTO/Al/HfO_x_ cascaded heterojunctions is located at 220 nm, which can be attributed to the superposition of the optical responses of GTO and HfO_x_. The GTO/Al/HfO_x_ cascade heterojunctions exhibit superior performance metrics compared to previously documented state-of-the-art DUV photoconductive sensors^21^, photovoltaic sensors^14,15^, phototransistors, and avalanche diodes (see Supplementary Table S2 for details). With a high detectivity of 2.9 × 10^16^ Jones and a peak external quantum efficiency of 7.3 × 10^5^% (Fig. 1g), these results indicate a highly effective suppression of carrier recombination and a significant gain within the GTO/Al/HfO_x_ cascade heterojunctions.

### Photoresponse mechanism of GTO/Al/HfO_x_ cascade heterojunctions

**Fig. 2:**
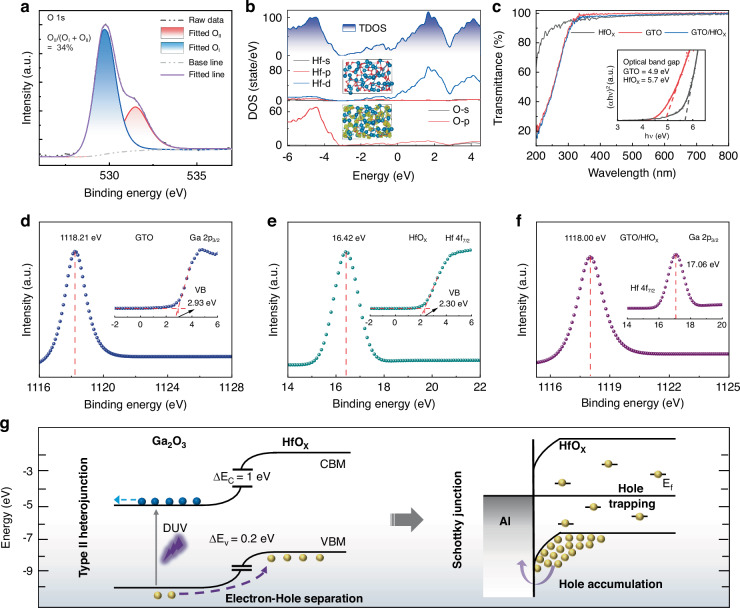
Photoresponse mechanism in GTO/Al/HfO_x_ cascade heterojunctions. **a** O 1 s XPS spectrum for HfO_x_ films. **b** Total and atom-projected partial density of states (DOS) for Hf and O in oxygen-deficient Hf_48_O_48_, as determined by ab initio molecular dynamics simulations. Insets are the computed amorphous structure and partial charge distribution, where the yellow bubbles denote regions of increased charge. **c** Transmission spectra of the GTO, HfO_x_, and GTO/HfO_x_ heterojunction films, with insets showing the Tauc plot used to determine the optical band gaps of the GTO and HfO_x_ films. **d** Ga 2p_3/2_ XPS and valence band spectra of the GTO film. **e** Hf 4f_7/2_ XPS and valence band spectra of the HfO_x_ film. **f** Ga 2p_3/2_ and Hf 4f_7/2_ XPS spectra of the GTO/HfO_x_ heterojunction with a 4 nm HfO_x_ layer. **g** Schematic representation of the calculated band structure for the cascade heterojunctions, comprising a type-II GTO/HfO_x_ heterojunction and an Al/HfO_x_ Schottky junction, illustrating the separation of photo-generated electron-hole pairs, with holes being blocked by the Schottky barrier and trapped by sub-band gap defects within the oxygen-deficient HfO_x_ layer

To delve into the mechanism behind the marked enhancement in the photoresponse brought about by the HfO_x_, we conducted X-ray photoelectron spectroscopy (XPS) characterization and density functional theory calculations to analyze the atomic and electronic configurations of the oxygen-deficient HfO_x_. The O 1 s XPS peak of the HfO_x_ was subjected to Gaussian fitting, which revealed two distinct peak positions (Fig. 2a). The peak O_I_, observed at the lower binding energy, corresponds to the lattice oxygen within the Hf-O bonds, while the peak O_II_ at the higher binding energy is indicative of oxygen vacancies^21^. The calculated area ratio of O_II_ to (O_I_ + O_II_) is about 34%, which signifies high oxygen vacancy defects within the HfO_x_ layer. Based on the observed variation in stoichiometric ratio, the total and partial density of states (DOS) for oxygen-deficient HfO_x_ system were calculated using ab initio molecular dynamics simulations, as shown in Fig. 2b and Supplementary Fig. S2. The Hf_48_O_48_ is replete with oxygen vacancies and hafnium dangling bonds, causing the hafnium atoms to migrate towards these vacancy sites, thus giving it an amorphous appearance and asymmetric charge distribution (insets of Fig. 2b). In the total DOS, the defect-free Hf_48_O_96_ structure exhibits no mid-gap states, while Hf_48_O_72_ possesses defect states of intermediate density and the Hf_48_O_48_ has numerous defect states at deeper energy levels stemming from the 5 d orbital interactions of hafnium atoms. These defect states were introduced below the CBM and even near the VBM. These findings are in alignment with the transmission spectra of the GTO heterojunction film, which demonstrates deep-level defect absorption for light with wavelengths extending to 400 nm, as depicted in Fig. 2c.

Next, the energy band alignment at the GTO/HfO_x_ heterojunction interface was identified by using XPS valence band spectra measurements:

1 $$\Delta {E}_{V}=({E}_{Ga2{p}_{3/2}}^{{GTO}}-{E}_{VBM}^{GTO})-({E}_{{\rm{Hf}}4{f}_{7/2}}^{Hf{O}_{x}}-{E}_{VBM}^{Hf{O}_{x}})-({E}_{CL}^{GTO}(i)-{E}_{CL}^{Hf{O}_{x}}(i))$$

2 $$\Delta {E}_{C}={E}_{g}^{G{TO}}-{E}_{g}^{Hf{O}_{{\rm{x}}}}-\Delta {E}_{V}$$

Where the ∆*E*_C_ and ∆*E*_V_ represent the conduction and valence band offset at the GTO/HfO_x_ heterojunction interface, the ${E}_{Ga2{p}_{3/2}}^{G{T}O}$ and ${E}_{VBM}^{G{TO}}$ are the binding energy of Ga 2p_3/2_ XPS peak and the VBM of the GTO film, the ${E}_{{\rm{Hf}}4{f}_{7/2}}^{H{\rm{f}}{O}_{x}}$ and ${E}_{VBM}^{H{\rm{f}}{O}_{x}}$ are the binding energy of Hf 4f_7/2_ XPS peak and the VBM relative to Fermi level of the HfO_x_ film, the ${E}_{CL}^{G{TO}}({i})$ and ${E}_{CL}^{H{\rm{f}}{O}_{x}}(i)$ are the binding energy of the Ga 2p_3/2_ and Hf 4f_7/2_ XPS peaks of the GTO/HfO_x_ heterojunction, the ${E}_{g}^{G{TO}}$ and ${E}_{g}^{Hf{O}_{{\rm{x}}}}$ are the optical bandgap of GTO and HfO_x_ films, respectively. As shown in the Fig. 2d−f, the ${E}_{Ga2{p}_{3/2}}^{G{T}O}$ and ${E}_{VBM}^{G{TO}}$ are 1118.2 and 2.93 eV, the ${E}_{{\rm{Hf}}4{f}_{7/2}}^{H{\rm{f}}{O}_{x}}$ and ${E}_{VBM}^{H{\rm{f}}{O}_{x}}$ are 16.42 and 2.30 eV, the ${E}_{CL}^{G{TO}}({i})$ and ${E}_{CL}^{H{\rm{f}}{O}_{x}}(i)$ are 1118.00 and 17.06 eV. The ${E}_{g}^{G{TO}}$ and ${E}_{g}^{Hf{O}_{{\rm{x}}}}$ were calculated to be 4.9 eV and 5.7 eV using Tauc’s method (inset of Fig. 2c), and the $\Delta {E}_{V}$ and $\Delta {E}_{C}$ were 0.22 and 1.02 eV, respectively. To further elucidate the interfacial energy band alignment, we performed ultraviolet photoelectron spectroscopy measurements on GTO and HfO_x_ films. As shown in Supplementary Fig. S3, the Fermi levels of GTO and HfO_x_ were determined to be 5.90 eV and 6.26 eV, respectively. Additionally, deep-level defect states within HfO_x_ were observed at 1.5 eV and 2.2 eV above the valence band maximum. Based on the above calculations and the work function of the Al electrodes, we illustrate the energy band structure of the GTO/Al/HfO_x_ cascade heterojunctions in Fig. 2g, and proceed to describe the dynamics of the photogenerated carriers as follows.

Upon exposure to DUV radiation, the inherent electric field at the GTO/HfO_x_ interface promotes the separation of photogenerated electrons and holes. Electrons traverse the GTO layer and are efficiently collected by the Al electrode, while the holes migrate to the HfO_x_ layer. Then a large number of holes become trapped by deep-level defects within the HfO_x_ layer, as evidenced by the gradual decay of the surface potential difference across the GTO/Al/HfO_x_ cascade heterojunctions in-situ measured after DUV light illlumination (Supplementary Fig. S4). The promotion of hole trapping by the Al/HfO_x_ hole Schottky barrier consists with the reduced responsivity observed in the GTO/Au/HfO_x_ heterojunction, which features a lower hole barrier at the Au/HfO_x_ interface (see Supplementary Figs. S5–7 for details). The trapped holes in the HfO_x_ layer generate a Coulombic force that extracts electrons into the GTO layer from the Al electrode. Electrons undergo multiple cycles within the lifetime of the trapped holes, leading to a prolonged enhancement in photocurrent that continues until the holes recombine with electrons. This process is akin to the injection of multiple electrons from a single photon event and thus leading to large responsivity.

### Optoelectronic synaptic plasticity of GTO/Al/HfO_x_ cascade heterojunctions

**Fig. 3:**
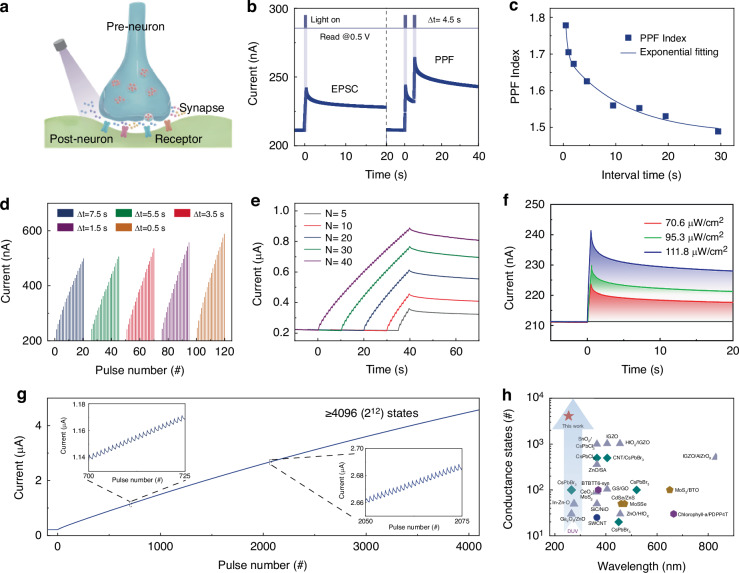
Optoelectronic synaptic plasticity in GTO/Al/HfO_x_ cascade heterojunctions. **a** Schematic diagram of the biological synapse controlled by light stimulations. **b** Typical EPSC and PPF functions realized by a single DUV spike (111.8 μW/cm^2^, 0.5 s) and pair of spikes (Δt = 4.5 s), respectively. **c** The variation of PPF versus the relative spike timing. The photocurrent variation obtained under a DUV spike train with **d** different interval times, **e** different spike numbers, and **f** different light intensities. **g** Conductance states under 4096 times of input optical pulses. Insets show the randomly enlarged graphs of response curve. **h** Compared number of conductance states and photoresponse wavelengths among optoelectronic synapses prepared by oxide^17,30–38^, organic^4,39^, two-dimensional^40–42^, carbon^43,44^, and perovskite^45–49^ materials

NVS integrates the multifunctionality of image sensing, storage, and processing, which can eliminate the redundant data and high-power consumption in separate architecture^22^. The GTO/Al/HfO_x_ optoelectronic synapse with high responsivity provides ideal hardware to emulate a biological synapse for NVS. As illustrated in Fig. 3a, light stimulations can strengthen the synaptic weight between the pre- and post-neurons, a phenomenon known as optoelectronic synaptic plasticity, which was reproduced in GTO/Al/HfO_x_ optoelectronic synapse. Figure 3b shows the photo response under the action of the DUV spike. The illumination duration is 0.5 s, and the photocurrent evolution is monitored with a voltage bias of 0.5 V. An abrupt increase in photocurrent (~32 nA) was obtained by applying a single DUV spike. The photocurrent decays to an intermediate state after the DUV spike is removed, which resembles to the excitatory postsynaptic current (EPSC) behaviors of biological synapse^23,24^. As shown in Fig. S8, the response time of the EPSC in our device can reach 10 ms, which can be comparable with that of human vision (ranging from 40 ms to 150 ms). Besides that, the photocurrent can be further enhanced using successive DUV spikes, before decaying to the initial state. The above behavior is similar to paired-pulse facilitation (PPF)^25,26^. In particular, the interval between two spikes dominates the facilitation, in which a shorter interval corresponds to greater enhancement, as shown in Fig. 3c. Such PPF functions can be extended to a spike-rate-dependent plasticity (SRDP) function through the use of spike trains, as shown in Fig. 3d. After applying a spike train with 15 consecutive DUV spikes, the EPSCs increase from 0.487 to 0.588 μA, when the interval time decreases from 7.5 to 0.5 s. The larger EPSCs can be obtained by increasing the spike rate, which is similar to the SRDP function of biological synapses^27^. Furthermore, the stimulus number is also a significant factor to modulate biological synaptic plasticity. As depicted in Fig. 3e, the photocurrent change exhibits a more obvious increase with more DUV spikes.

Intensity-dependent plasticity is considered as necessary characteristic to perceive complicated image information^28,29^. Figure 3f shows the evolution of photocurrent under optical stimulus of different intensities. The current output from lower light intensity exhibits faster decay, compared with that from higher light intensity. Beyond the above synaptic plasticity, the high linearity and multi-conductance states are imperative for high-precision neuromorphic computing^30^. Herein, the optical signals are optimized to investigate the quantity of conductance states. Both the irradiation duration and interval time are set as 100 ms, and optical intensity is fixed. Corresponding photocurrent response under the action of periodical irradiation for 4096 times is plotted in Fig. 3g. The insets demonstrate the specific response curve at different times, in which stepwise photocurrent increases are obtained. Herein, the optical signal parameters were carefully optimized to maximize the number of conductance states while maintaining high conductance modulation linearity and distinguishable conductance states, which are crucial for achieving high-accuracy image recognition. Although the high sensitivity of our GTO/Al/HfO_x_ cascade heterojunctions optoelectronic synapses allows for shorter irradiation durations and interval times to achieve finer resolution of conductance states, a trade-off exists between the number of conductance states and the linearity of conductance modulation (Supplementary Fig. S9). This trade-off restricts the optimal irradiation duration and interval time to 100 ms. In order to benchmark the performance of the GTO/Al/HfO_x_ cascade heterojunctions, the quantity of conductance states is compared with those of other materials, including oxide^17,30–38^, organic^4,39^, two-dimensional^40–42^, carbon^43,44^, and perovskite^45–49^ materials. As depicted in Fig. 3h, our device presents the highest quantity of conductance states. Meanwhile, the response wavelength in DUV region enables to realize image perception with resistance to visible light interference. The outstanding performances of our device provide the foundation for high-precision neuromorphic computing and visual perception.

### Application of optoelectronic synapse in logic operation and fingerprint classification

**Fig. 4:**
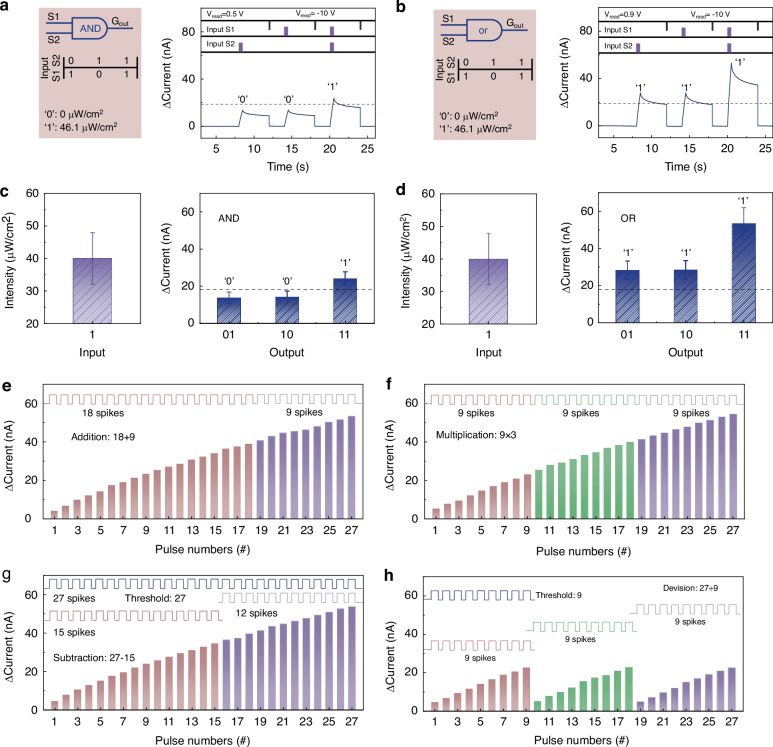
Logic operation and arithmetic computing in GTO/Al/HfO_x_ optoelectronic synapse. **a**, **b** AND and OR logic operations by using two DUV spikes (0 μW/cm^2^, 46.1 μW/cm^2^, respectively). The device is restored to an initial state using electrical pulse (−10 V, 50 ms) after each logic operation. **c**, **d** AND logic and OR logic operation by using two presynaptic spikes with 40% amplitude fluctuation, namely, the light intensity of DUV spike ranges from 35 to 53.5 μW/cm^2^. **e, f, g, h** Arithmetic computing of addition, multiplication, subtraction, and division demonstrated by utilizing the examples of 18 + 9, 9 × 3, 27–15, and 27 ÷ 9, respectively

The logic operation functions with fault-tolerance capability and arithmetic computing have been implemented in GTO/Al/HfO_x_ optoelectronic synapses. In the proposed logic operation, a pair of DUV spikes (0.5 s) from two independent optical inputs to control the output photocurrent signals. The “light on” and “light off” represent the logic input of “1” and “0”, respectively. A bias voltage of 0.5/0.9 V is selected to monitor the evolution of photocurrent change for AND/OR operation. The photocurrent threshold is set as 18.8 nA. As demonstrated in Fig. 4a, logical output “1” (above the threshold of 18.8 nA) is obtained only when both the optical inputs are present. Otherwise, the output result is logical “0”, which is below the threshold photocurrent. Both the AND/OR operations have the same threshold of 18.8 nA. It is worth noting that the device will be restored to its initial state using electrical pulse (−10 V, 50 ms) after each logic operation. As for the “OR” logic operation, a single DUV spike can induce the photocurrent above the threshold, resulting in logic result “1” (Fig. 4b). Furthermore, the logic operation function in our device exhibits excellent fault-tolerance capability. As shown in Figs. 4c and 4d, the input signals with an intensity fluctuation of 40% are applied to the device. The logic output results are still valid for the “AND” and “OR” logic operations, despite the obvious fluctuates. On the other hand, optoelectronic memristive behavior with high linearity is indispensable to implement arithmetic computing functions. As shown in Supplementary Fig. S9, the photocurrent change induced by *n* optical spikes is defined as *ΔI*_n_. The value of *ΔI*_n_ reaches 53.35 nA under the irradiation of 27 successive spikes, indicating a linear increase with the quantity of optical spikes. Then the arithmetic computing of addition is demonstrated by applying two stimulation sequences, as shown in Fig. 4e. The *ΔI*_n_ of 53.35 nA is obtained with the sequence of 18 spikes followed by another sequence of 9 spikes. The output result is equivalent to the *ΔI*_n_ of 27 successive spikes, achieving the addition of 18 + 9 = 27. The same *ΔI*_n_ can be obtained by using the stimulation sequences with opposite order, which is the commutative law of addition (18 + 9 = 9 + 18 = 27, Supplementary Fig. S10). As for the arithmetic computing of multiplication, three sequences of 9 spikes are applied in turn to achieve the value of *ΔI*_27_, i.e., the multiplication of 9×3 = 27. The commutative law of multiplication is verified by the equal result from nine sequences of three spikes. Furthermore, an additional 12 optical spikes are required to achieve the value of *ΔI*_27_, after applying 15 successive spikes. Hence, the arithmetic subtraction of 27−12 = 15 is obtained. Figure 4f demonstrates the arithmetic computing of division, in which the *ΔI*_9_ is selected as threshold for divisor. The quotient will increase by 1, when the photocurrent change reaches the threshold of *ΔI*_9_. If the successive spikes cannot trigger the threshold, the spike number will be recorded as remainder. Therefore, the quotient is 3 and the remainder is 0 for the division of 27 ÷ 9. The above arithmetic computing of addition, subtraction, multiplication, and division verifies the stable optoelectronic memristive behaviors.

**Fig. 5:**
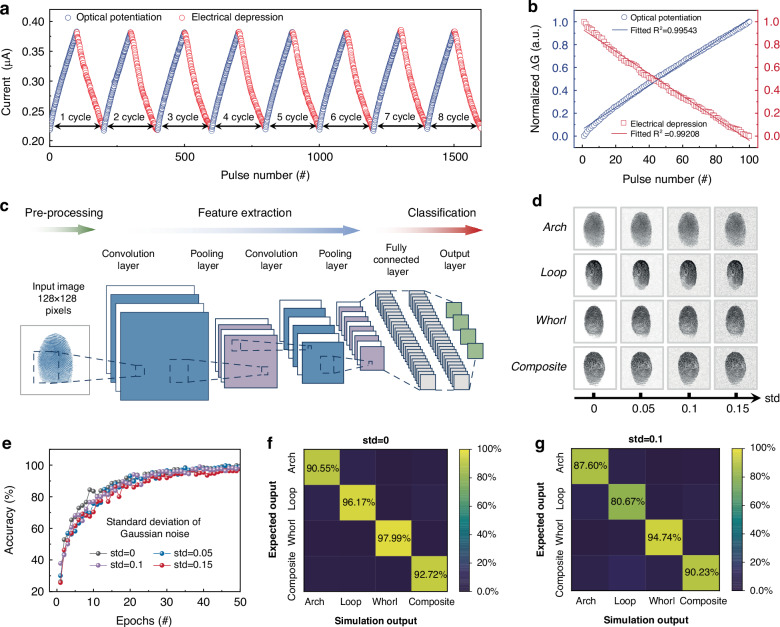
Linear optical potentiation/electrical depression behaviors and high-accuracy image classification functions. **a** Eight cycles of optical potentiation/electrical depression behaviors. **b** Typical cycle of optical potentiation/electrical depression behaviors under consecutive training pulses, presenting a high linearity of weight updating. **c** Schematic illustration of stimulated NVS for the application of fingerprint classification. **d** The fingerprint images with different-level Gaussian noise, whose standard deviation (std) ranges from 0 to 0.15. **e** Recognition accuracy evolution with training epochs for the NVS with different noise ratios. The classification output results depicted in the confusion matrixes (**f**) without and **g** with Gaussian noise (std is set as 0.1)

Optical potentiation and electrical depression behaviors were investigated to implement high-level image processing function. In particular, the linearity and symmetricity of synaptic weight update is the primary factor to dominate the overall accuracy in NVS^50^. Figure 5a shows periodic optical potentiation (100 pulses with width of 50 ms) and electrical depression (100 pulses with a width of 50 ms) behaviors. The GTO/Al/HfO_x_ optoelectronic synapses exhibit negligible fluctuation during the 8-cycle test, indicating outstanding stability. Furthermore, both the optical potentiation and electrical depression behaviors are highly linear, as shown in Fig. 5b. Herein, the linearity of synaptic weight update is evaluated by the determination coefficient R^2^ in linear fitting^3^. The dashed lines represent the linear fitting, in which the R^2^ is 0.995 and 0.992 for optical potentiation and electrical depression behaviors, respectively. In addition to high weight update linearity, the GTO/Al/HfO_x_ optoelectronic synapse also possessed high uniformity and stability towards device-to-device variability (Supplementary Fig. S11). The obtained standard deviations (σ) to mean value (µ) ratios were all below 10%, demonstrating high uniformity and stability. The excellent linear correlation and high stability of conductance variation behaviors enable to simplify the backpropagation process and improve convergence speed.

Based on the experimentally obtained parameters of the GTO/Al/HfO_x_ optoelectronic synapse array, a stimulated NVS is constructed to realize fingerprint classification. The DUV stimulation exhibits outstanding advantages to reveal more intricate details due to its shorter wavelength, and the DUV light can make the latent fingerprints visible without damaging the evidence^9^. Herein, four types of fingerprints (arch, loop, whorl, and composite patterns) are chosen from the Fingerprint Verification Competition (FVC 2002) database. 80% of the fingerprint images were designated as the training set, while the remaining 20% were allocated for the testing set (namely, the unseen images). As shown in Figs. 5c and 5d, the input fingerprint image with pixels of 128×128 is used to demonstrate the feasibility of images classification functions in stimulated NVS constructed by the GTO/Al/HfO_x_ optoelectronic synapses. The convolutional neural network (CNN) is composed of two convolution layers with two pooling layers and two fully connected layers. Herein, the convolution layers with pooling layers are used for feature extraction, and the fully connected layer acts for information classification, respectively. Then output current can be obtained by utilizing the weight-sum operation between the hidden and output layers. The synaptic weight is updated to minimize the output error, which follows the long-term potentiation and depression behaviors. The softmax function is selected as the activation function. The backpropagation algorithm is utilized to update the synaptic weight, which follows the long-term potentiation and depression behaviors. As depicted in Fig. 5e, the recognition accuracy during training can reach 99.6% after just 50 training epochs. The classification output results are depicted in the confusion matrixes of the fingerprint recognition with the unseen images as the test set (Fig. 5f), in which the statistics values in matrix diagonals represent the correct classification of each fingerprint type. Taking Whorl type as an example, the testing accuracy is around 98%, suggesting that the GTO/Al/HfO_x_ NVS possesses the ability to achieve high-efficient fingerprint classification. Herein, we also investigated the influence of device-to-device variability on the recognition accuracy of the stimulated NVS, in which the synaptic weight randomly follows one of 16 long-term potentiation and depression curves from 16 devices. As shown in Fig. S12, although the recognition accuracy decreases from 99.6% to 95.3% after just 50 training epochs and the testing result of classification decreases from 98% to 89%, the experimental results remain the high performance, highlighting the robustness of our system. Additionally, we also investigate the fault-tolerance capability of our NVS to mimic the potential optical interference. Figure 5d presents fingerprint images with different Gaussian noise levels, whose standard deviation (std) ranges from 0 to 0.15. The classification output results with random noise (std = 0.1) are depicted in the confusion matrixes of Fig. 5g. Although the test accuracy decreases compared with the fingerprint without noise, the testing result reaches 94.7% with std of 0.1. Moreover, the final testing accuracy still exceeds 93.4%, even for Gaussian noise levels with std of 0.15 (Supplementary Fig. S13), indicating that our NVS enables us to realize high-precision image processing with fault-tolerance characteristics.

## Discussion

In conclusion, this work introduces a multistate and linearly programmable optoelectronic synapse, fabricated through the GTO/Al/HfO_x_ cascade heterojunctions design. Insights into the photoresponse performance and underlying mechanism suggest that the gain of the GTO/Al/HfO_x_ optoelectronic synapse is mainly controlled by the built-in-field-assisted electron-hole separation at the GTO/HfO_x_ interface, coupled with the subsequent Al/HfO_x_ Schottky barrier-mediated trapping of holes in the oxygen-deficient HfO_x_ layer. This synergy yields ultrahigh responsivity. The GTO/Al/HfO_x_ optoelectronic synapses have achieved unprecedented 4096 conductance states and excellent conductivity regulation linearity with a fitting coefficient of 0.992, enabling them to simulate biological behaviors and perform optoelectronic logical operations and arithmetic computing. Furthermore, the function of fingerprint classification with high accuracy has been demonstrated with GTO/Al/HfO_x_ NVSs. These exceptional capabilities demonstrate that our proposed cascade heterojunctions strategy is effective in enhancing the performance of ultra-wide bandgap semiconductor optoelectronic synapses for DUV NVSs and has the potential for application to a broader range of materials.

## Materials and methods

### Preparation of GTO films

The GTO layer was deposited on a c-plane sapphire substrate using the sol-gel method. The substrate was initially cleaned with deionized water, acetone, and ethanol. The GTO precursor was synthesized from gallium nitrate hexahydrate (as the gallium source), ethylene glycol (solvent), triethanolamine (stabilizer), and 99.99% tin foil (tin dopant). The Ga^3+^ concentration of the precursor was 0.8 mol L^‒1^ and the tin doping concentration was 13%. The mixture was stirred at 60 °C for 2 hours and then aged at room temperature for 24 hours. The precursor was spin-coated onto an oxygen-plasma treated sapphire substrate at 3000 rpm for 30 seconds, followed by a 10-minute solvent evaporation step at 100 °C on a hotplate. This process was repeated six times, with each layer air-dried at 500 °C for 15 minutes. The final samples were annealed at 600 °C in air for 2 hours. For comparative purposes, pure Ga_2_O_3_ films were also prepared using an identical procedure.

### Preparation of HfO_x_ films

Initially, either Al or Au interdigital electrodes, each 1000 µm long and 30 µm wide with a 70 µm gap, were evaporated onto the GTO films to fabricate a metal-semiconductor-metal structured DUV photosensor. Each device featured 10 pairs of these electrodes. Subsequently, the HfO_x_ layer was deposited on the GTO/Al surface using a magnetron sputtering method with the aid of a mechanical mask. The HfO_x_ target, supplied by Zhongnuo New Materials (Beijing) Technology Co., was of 99.99% purity. The deposition of the HfO_x_ film occurred under a base chamber vacuum of 5.0 × 10^‒6^ Torr, a glow pressure of 0.05 Torr, a growth pressure of 7.5 × 10^‒3^ Torr, an argon flow rate of 18.0 sccm, and a sputtering power of 60 W, all at ambient temperature. The HfO_x_ films for all GTO/Al/HfO_x_ devices were deposited under an oxygen partial pressure of 0%.

### Materials characterization and device measurements

The micro-structure of the GTO and HfO_x_ films was evaluated by the Rigaku D/max-2500 XRD employing Cu Ka radiation and TEM (FEI Tecnai TF-20) coupled with energy dispersive spectrometers. The surface characteristics were probed through AFM (Bruker). KPFM was conducted in an in situ manner to monitor the films’ response to DUV light, employing amplitude modulation of the tapping mode with a Pt/Ir tip. Chemical composition analysis was achieved through XPS (VG ESCALAB LKII) using a Mg KR-ADES source. Optical transmission properties were measured with a Hitachi UH4150 spectrometer, with calibration against a sapphire. Electrical measurements, including time-dependent current and voltage-current curves, were obtained using a Keithley 4200A-SCS parameter analyzer under both illuminated and dark conditions. The light source was a UV-enhanced xenon lamp coupling to a monochromator.

### DFT calculations

First-principles calculations were carried out using the Vienna Ab Initio Simulation Package (VASP). The exchange-correlation functional was described by the generalized gradient approximation (GGA) within the Perdew-Burke-Ernzerhof (PBE) formulation. The amorphous structures of Hf_48_O_48_, Hf_48_O_72_, and Hf_48_O_96_ were generated through ab initio molecular dynamics (AIMD) simulations. During the AIMD simulations, the structures were equilibrated for 3 ps at 10 evenly spaced temperatures ranging from 2000 to 300 K, employing the Nosé thermostat to maintain the NVT ensemble. The AIMD simulations utilized a kinetic energy cutoff of 350 eV and a single Γ point to sample the Brillouin zone. The lattice parameters and atomic positions were fully relaxed until the force on each atom was less than 0.05 eV/Å. For self-consistent calculations, a cut-off energy of 450 eV was set for the plane wave basis, and a 3 × 3 × 2 Monkhorst-Pack mesh was employed. The lattice parameters and atomic positions were relaxed until the force on each atom was less than 0.02 eV/Å, with the convergence threshold for the energy in the self-consistent calculation set at 10^‒5^ eV.

## Supplementary information

Ultra-Highly Linear Ga_2_O_3_-based Cascade Heterojunctions Optoelectronic Synapse with Thousands of Conductance States for Neuromorphic Visual System

## Data Availability

The data that support the findings of this study are available from the corresponding author upon reasonable request.
