# Supplementary material for: Ultra-highly linear Ga2O3-based cascade heterojunctions optoelectronic synapse with thousands of conductance states for neuromorphic visual system
Source: Light Sci Appl. 2025 Sep 30;14:354. doi: 10.1038/s41377-025-01897-9 (PMC12485082; doi:10.1038/s41377-025-01897-9)
Supplement: Supplementary file 1 — Ultra-Highly Linear Ga2O3-based Cascade Heterojunctions Optoelectronic Synapse with Thousands of Conductance States for Neuromorphic Visual System [file 41377_2025_1897_MOESM1_ESM.docx]

Supporting Information

Ultra-Highly Linear Ga_2_O_3_-based Cascade Heterojunctions Optoelectronic Synapse with Thousands of Conductance States for Neuromorphic Visual System

Peng Li, Xuanyu Shan, Ya Lin*, Yi Du, Jiangang Ma, Zhongqiang Wang*, Xiaoning Zhao, Ye Tao, Haiyang Xu* and Yichun Liu

P. Li and X. Shan contribute equally to this work.

Key Laboratory for UV Light-Emitting Materials and Technology of Ministry of Education, Northeast Normal University, 5268 Renmin Street, Changchun 130024, China

E-mail: liny474@nenu.edu.cn; wangzq752@nenu.edu.cn; hyxu@nenu.edu.cn.


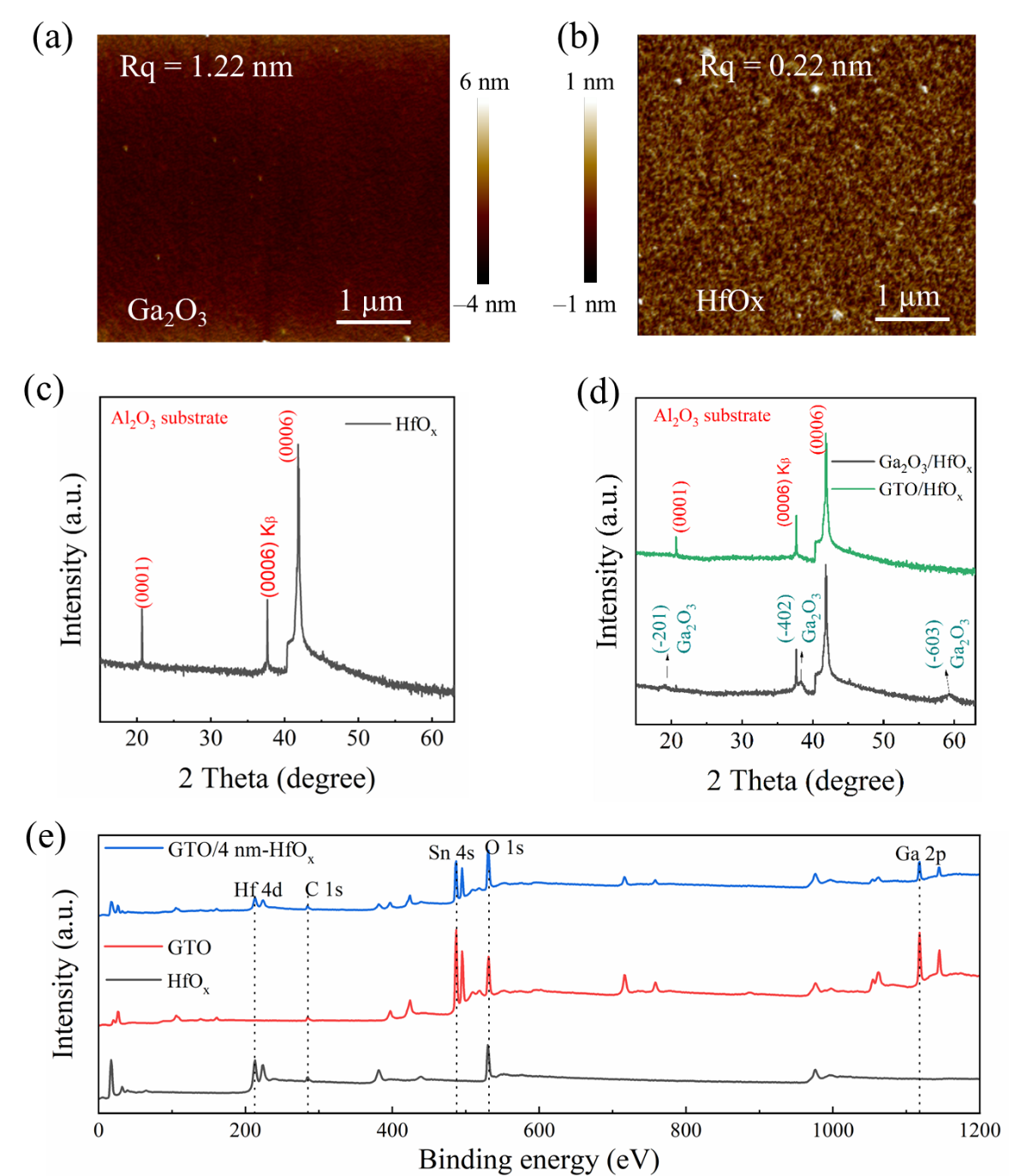


Figure S1. Characterization of Ga_2_O_3_, GTO and HfO_x_ films. (a) AFM image of the pure Ga_2_O_3_ film without tin doping. (b) AFM image and (c) XRD pattern of the HfO_x_ film on the sapphire substrate. (d) XRD patterns of the Ga_2_O_3_/HfO_x_ and GTO/HfO_x_ heterojunctions.

Figure S1a presents an atomic force microscopy (AFM) image of a pure Ga_2_O_3_ film fabricated using the sol-gel method. The pure Ga_2_O_3_ film exhibits a root-mean-square surface roughness of 1.2 nm, which is slightly greater than that of the GTO film (0.24 nm, as shown in Figure 1d). This finding suggests that tin doping is advantageous for improving the surface topology of Ga_2_O_3_ films. Figure S1b is the AFM image of a HfO_x_ film deposited on a sapphire substrate via room-temperature magnetron sputtering. The as-deposited HfO_x_ film appears relatively smooth, with the presence of a few nanoparticles. The X-ray diffraction (XRD) pattern depicted in Figure S1c confirms that the HfO_x_ film is amorphous. The XRD patterns of the pure Ga_2_O_3_ film reveal weak diffraction peaks corresponding to the (−201), (−402), and (−603) planes, indicative of partial crystallinity in the pure Ga_2_O_3_ film (Figure S1d). Following the incorporation of tin, these diffraction peaks are no longer observed in the GTO/HfO_x_ heterojunction film. This suggests that the tin doping inhibits the crystallization of Ga_2_O_3_, which is favorable for the formation of smooth GTO/HfO_x_ heterojunction.


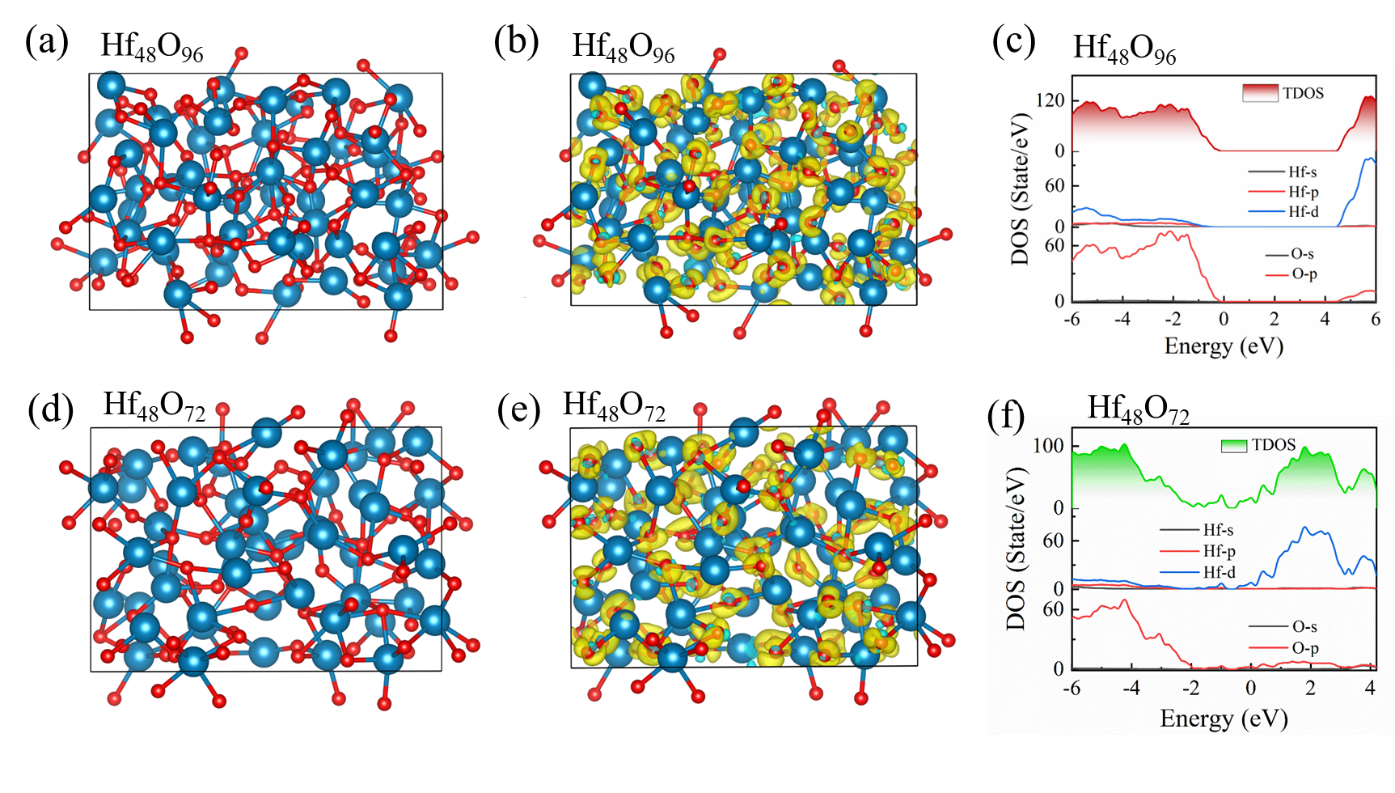


Figure S2. Molecular dynamics simulation of the atomic and electronic structure. Calculated (a) Hf_48_O_96_ structure and the corresponding (b) differential charge distribution. (c) Total (top panel) and atom projected partial density of states of Hf (middle) and O (bottom) species of Hf_48_O_96_. Calculated (d) Hf_48_O_72_ structure and the corresponding (e) differential charge distribution. (f) Total (top panel) and atom projected partial density of states of Hf (middle) and O (bottom) species of Hf_48_O_72_. The blue and red balls represent Hf and O atoms, respectively. The green and yellow bubbles indicate a decrease and increase in electron density.

To gain comprehensive understanding of how oxygen content influences the structure and electrical characteristics of amorphous HfO_x_, we conducted density functional theory calculations. We first investigated the stable amorphous configurations and partial charges of Hf_48_O_96_ and the oxygen-deficient Hf_48_O_72_, as illustrated in Figures S2a and 2d, respectively. The corresponding differential charges for the neutral Hf_48_O_96_ and oxygen-deficient Hf_48_O_72_ are presented in Figures S2b and 2e. The reduced coordination number of hafnium in Hf_48_O_72_, compared to Hf_48_O_96_, results in a relatively more relaxed structure and enhanced charge localization around the oxygen vacancy regions. The charge distribution within the neutral Hf_48_O_72_ is asymmetric, with negative charges (highlighted in yellow) clustering around the oxygen atoms. The defect-free Hf_48_O_96_ exhibits lower charge density and more uniform charge distribution. In contrast, some hafnium atoms near oxygen vacancies display uneven diffierential charge distributions due to their shift towards the vacancy sites. The partial and total density of states (DOS) for Hf_48_O_96_ and oxygen-deficient Hf_48_O_72_, shown in Figures S2c and 2f, reveal distinct differences in the sub-bandgap states. For Hf_48_O_96_, the theoretical optical bandgap of approximately 4.50 eV, as determined by the emergence of unoccupied density of states (DOS) above the Fermi level, is slightly lower than that observed in practical HfO_x_ films. This discrepancy is expected, as the commonly employed GGA functional in DFT simulations tends to underestimate bandgap values. Notably, additional defect states in the total DOS, primarily originating from the 5d-orbitals of hafnium, appear below the mid-gap. Consequently, the Fermi level of Hf_48_O_72_ upward shifted and intersects with defect level, leading to a decrease in the hole energy band. Furthermore, the number of defect states in the DOS of Hf_48_O_72_ is fewer than those in Hf_48_O_48_ (Figure 2b).


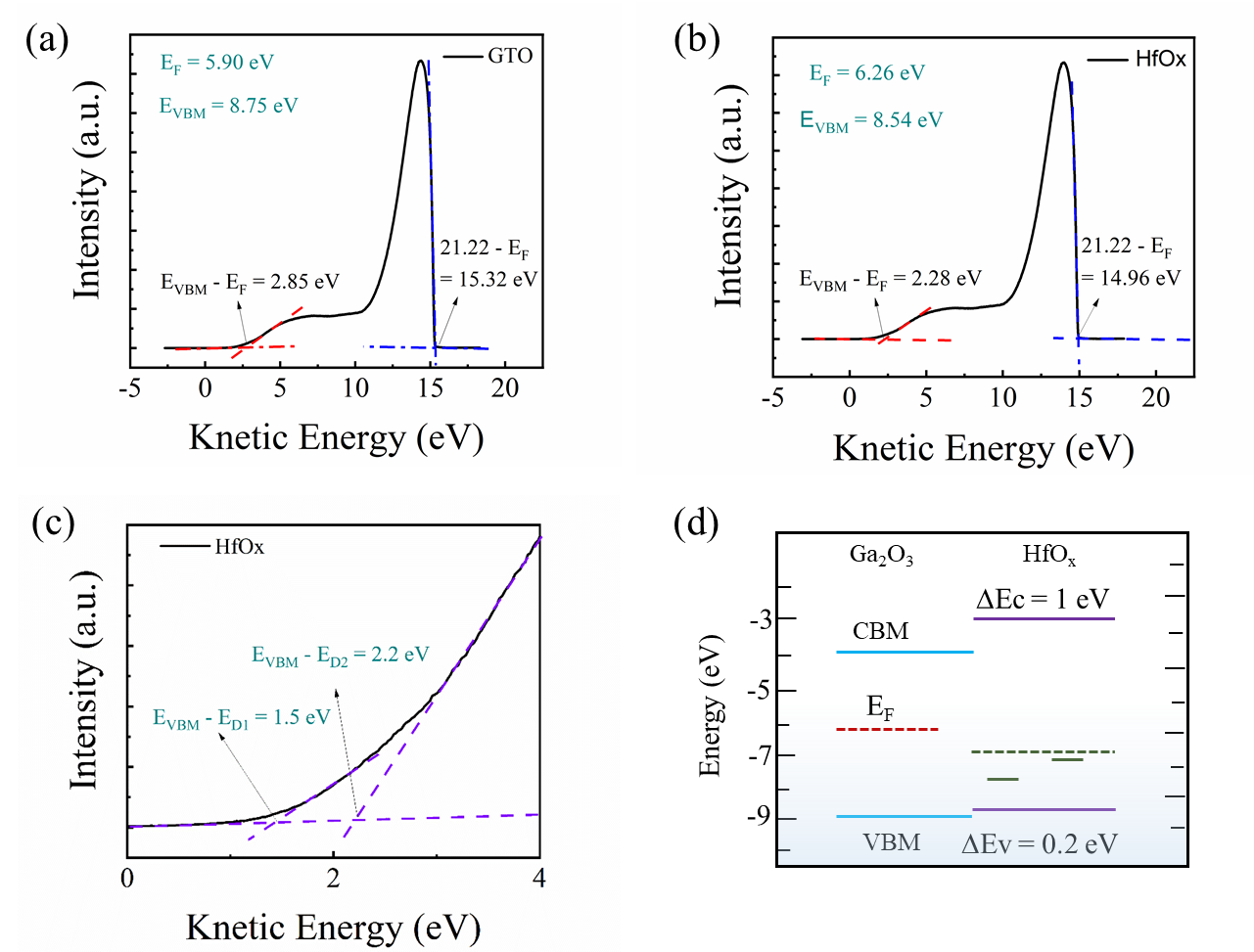


Figure S3. (a) Ultraviolet photoelectron spectroscopy (UPS) spectra of GTO film. (b) UPS spectra of HfO_x_ film. (c) Near valence region of HfO_x_ film, highlighting the energy positions of defect states relative to the valance band maximum. (d) Schematic energy level diagram illustrating the electronic structure of GTO and HfO_x_ prior to contact formation.


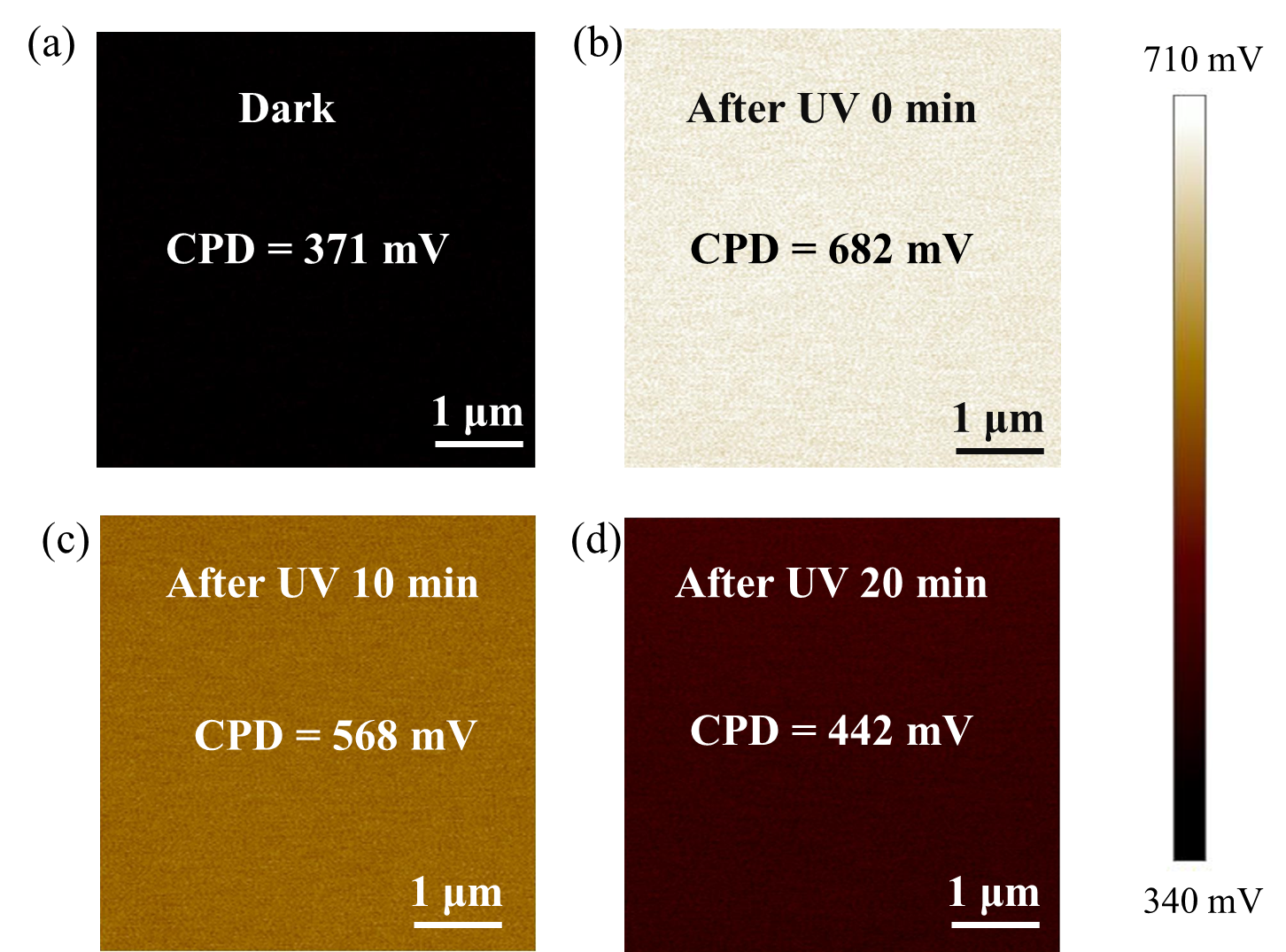


Figure S4. In-situ Kelvin probe force microscope (KPFM) analysis. Variations in surface potential of the GTO/Al/HfO_x_ cascade heterojunctions: (a) prior to, (b) immediately following DUV light exposure, (c) 10 minutes after DUV light illumination, and (d) 20 minutes post DUV light exposure.

In-situ KPFM measurements were carried out to examine the surface potential difference across the GTO/Al/HfO_x_ cascade heterojunctions during the transition from dark to illuminated conditions and its temporal evolution (Figure S4). In the absence of light, the surface potential difference was measured at around 371 mV. Upon exposure to light, the surface potential difference of the GTO/HfO_x_ heterojunction showed a general increase, indicative of light-induced hole trapping in defect states. After 20 minutes of irradiation, the surface potential difference stabilized at 442 mV. The gradual decay of the surface potential difference indicates the hole-trapping in the GTO/Al/HfO_x_ cascade heterojunctions.


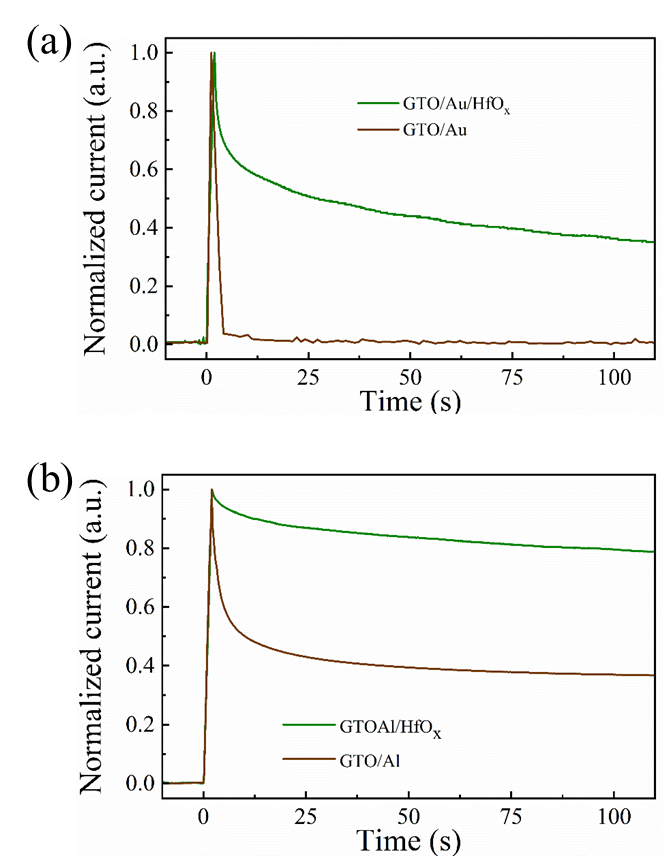


Figure S5. Normalized current versus time profiles (a) for the GTO/Au and GTO/Au/HfO_x_, and (b) for the GTO/Al and GTO/Al/HfO_x_ cascade heterojunctions optoelectronic synapses.

The excitatory postsynaptic current (EPSC) data for the GTO/Au and GTO/Au/HfO_x_ devices, as well as the GTO/Al and GTO/Al/HfO_x_ devices, have been normalized and are displayed in Figures S5a and b, respectively. The EPSC of the GTO/Au/HfO_x_ heterojunction maintained 35% of its peak value 100 s after the DUV light was turned off, while the GTO/Au device nearly reached 0%. Conversely, the EPSC of the GTO/Al/HfO_x_ cascade heterojunctions was at 79.54% of its peak value after 100 s, compared to 36.98% for the GTO/Al device. The comparison of EPSC values between devices equipped with identical (Al or Au) electrodes confirms that forming heterojunction with HfO_x_ enhances the memory capacity of GTO optoelectronic synapses. Furthermore, the EPSC comparison between devices with different (Al or Au) electrodes affirms that the established cascade heterojunctions further ensure the long-term retention properties of GTO/HfO_x_ heterojunction-based optoelectronic synapses.


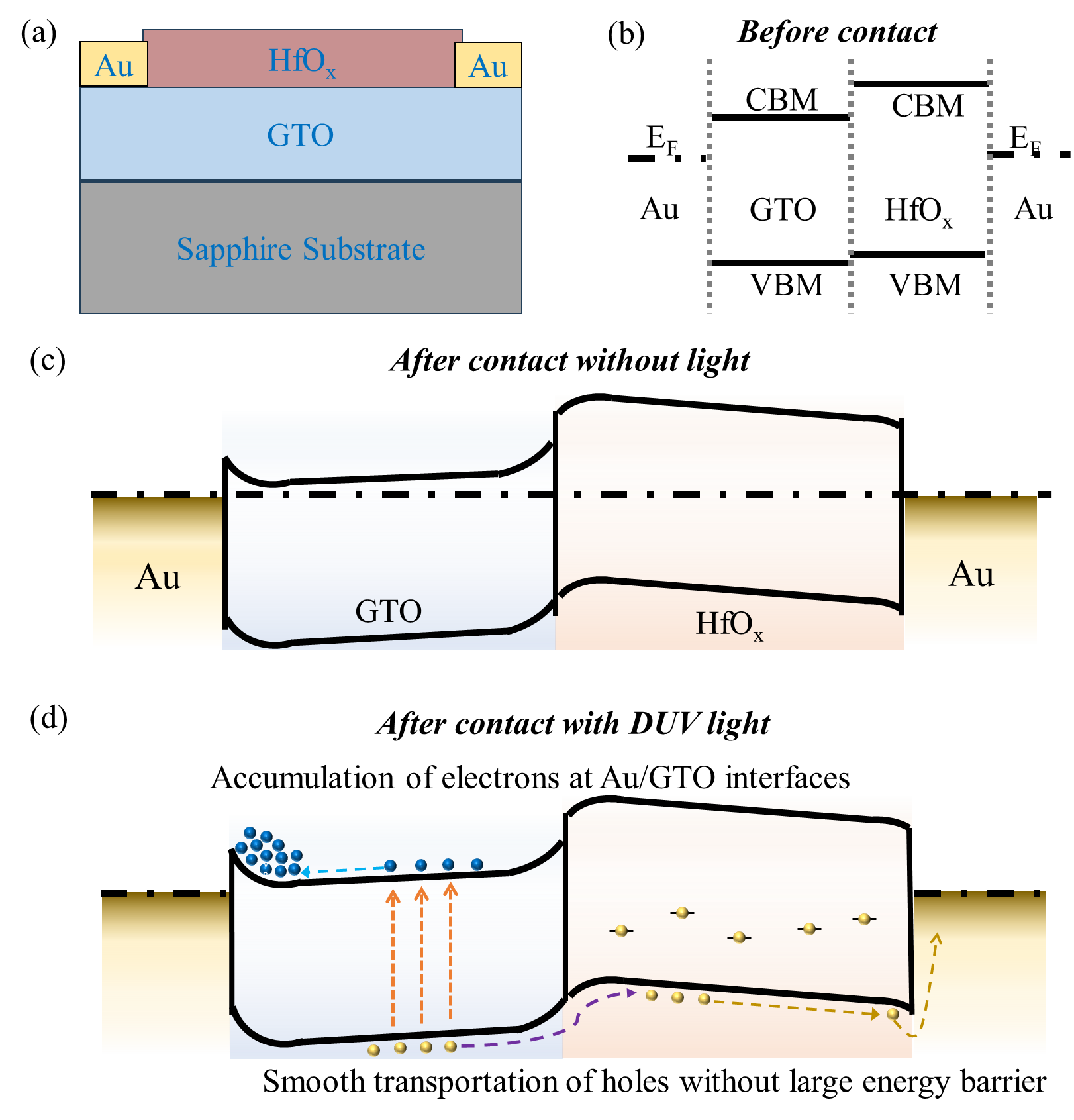


Figure S6. Mechanism of photoresponse in GTO/Au/HfO_x_ heterojunction. (a) Illustration of the GTO/Au/HfO_x_ structure. (b) Energy band alignment for the GTO/Au/HfO_x_ heterostructure prior to contact. CBM, VBM, and E_F_ denote the conduction band minimum, valence band maximum, and Fermi level, respectively. Energy band alignments of the GTO/Au/HfO_x_ heterostructure (c) in the absence of DUV light exposure, and (d) under DUV light irradiation.

Figure S6a and S6b depict the device architecture and the corresponding energy band alignment for the GTO/Au/HfO_x_ heterojunction. Under flat band conditions, GTO and HfO_x_ constitute a type II heterojunction with a relatively large conduction band offset. Upon establishing contact, three interfaces of Au/GTO, GTO/HfO_x_, and HfO_x_/Au are formed (Figure S6c). Upon irradiation with DUV light, photogenerated electron-hole pairs are produced within the GTO layer. Driven by the internal built-in electric field of the GTO/HfO_x_ heterojunction, holes migrate towards the HfO_x_ layer, while electrons are directed towards the GTO layer. Before being collected by the Au electrodes, a portion of the electrons in the GTO layer is impeded by the built-in field at the Au/GTO Schottky junction interfaces (Figure S6d). This obstruction enhances the recombination rate of electron-hole pairs, thereby hindering the increasement in the device’s responsivity.


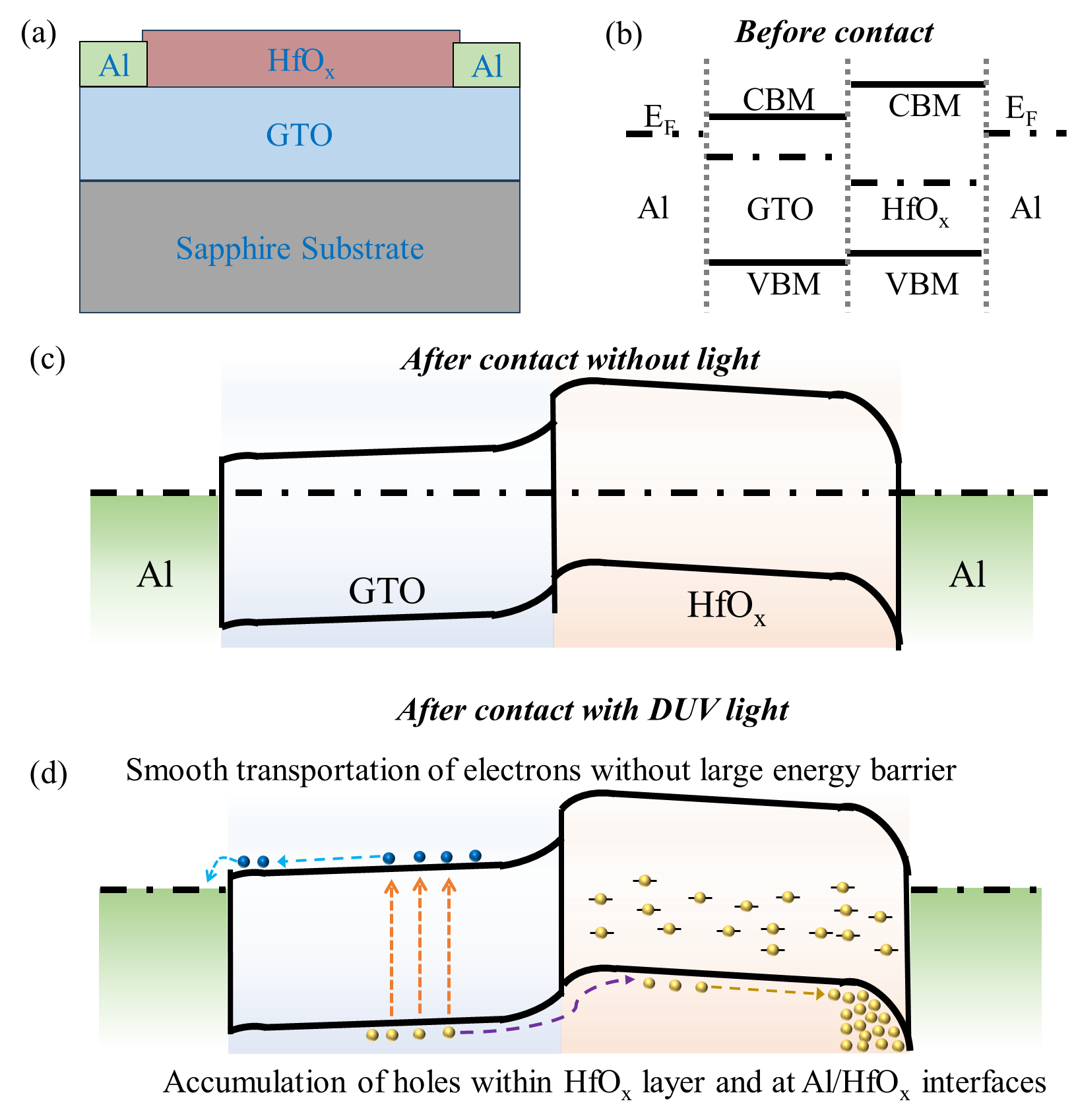


Figure S7. Mechanism of photoresponse in GTO/Al/HfO_x_ cascade heterojunctions. (a) Illustration of the stack structure. Energy band alignment (b) prior to contact, (c) in the absence of DUV light exposure, and (d) under DUV light irradiation.

Figure S7a and S7b illustrate the stack structure and the corresponding energy band alignment for the GTO/Al/HfO_x_ cascade heterojunctions. In the flat band scenario, the Fermi levels of both GTO and HfO_x_ are lower than that of Al. Upon forming a contact, the Al/HfO_x_ interface develops a high hole Schottky barrier, while the GTO/Al interface establishes a nearly Ohmic contact for electron transport (Figure S7c). When exposed to DUV light, photogenerated electron-hole pairs are created within the GTO layer (Figure S7d). Guided by the built-in electric field of the GTO/HfO_x_ heterojunction, holes move towards the HfO_x_ layer, and electrons flow towards the GTO layer, which is similar to the case with Au electrodes. The photogenerated electrons can traverse the GTO layer and reach the Al electrode without hindrance. However, a portion of the holes in the HfO_x_ layer is impeded by the internal built-in field at the GTO/Al Schottky junction interfaces. This barrier promotes the trapping of holes by the abundant defects present in the oxygen-deficient HfO_x_ layer. The recombination and annihilation rates of these oxygen vacancy-trapped holes with electrons are relatively slow in the HfO_x_ layer due to the higher energy barrier near these oxygen vacancy defects. These trapped holes act as a photogate, attracting multiple electrons to be injected into the GTO layer, thereby resulting in a substantial gain for the GTO/Al/HfO_x_ cascade heterojunctions. Notably, upon the absorption of a single photon, several electron cycles occur across the GTO layer. This gain mechanism leads to a continuously increasing photocurrent during the light irradiation until saturation, thereby enhancing the GTO/Al/HfO_x_ cascade heterojunctions’ responsivity.


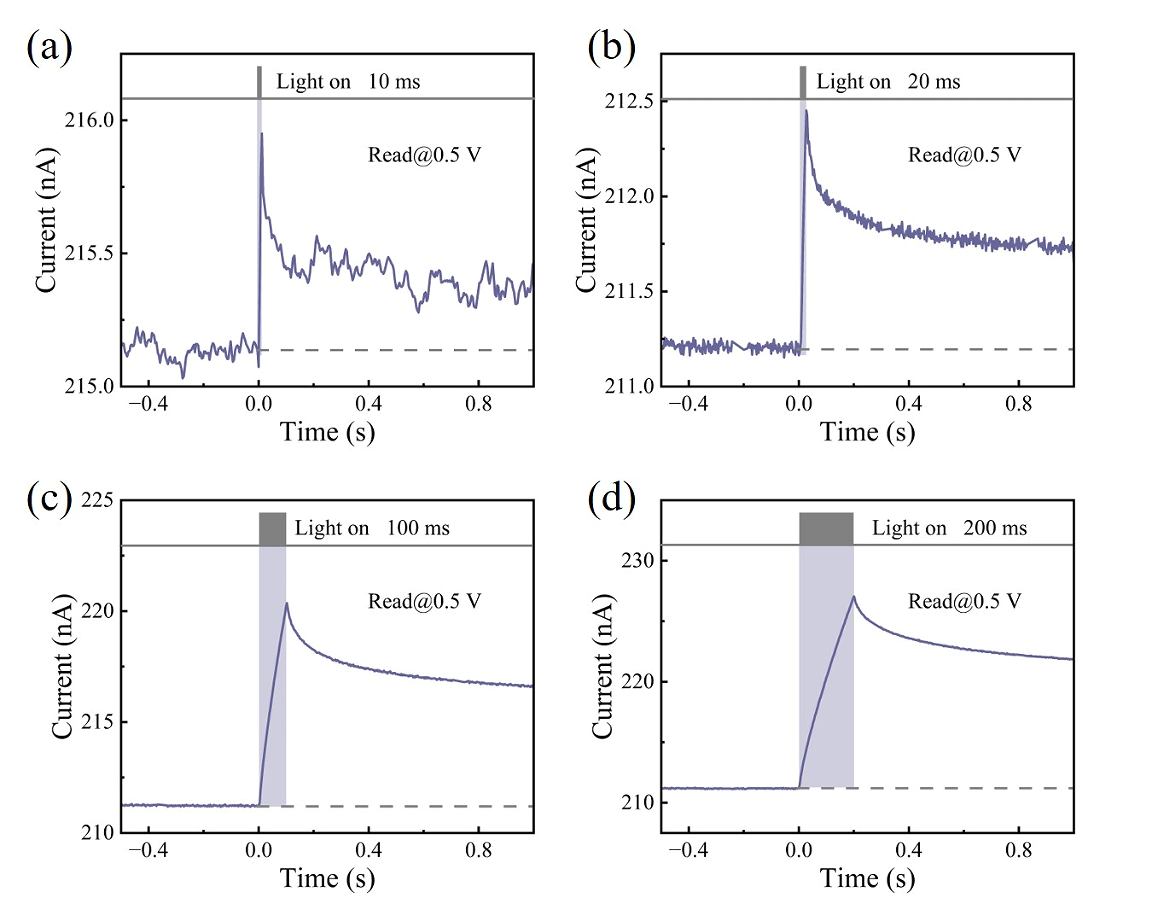


Figure S8. (a-d) The response current of the GTO/Al/HfO_x_ cascade heterojunctions under the light illumination with duration time decreasing from 200 ms to 10 ms.


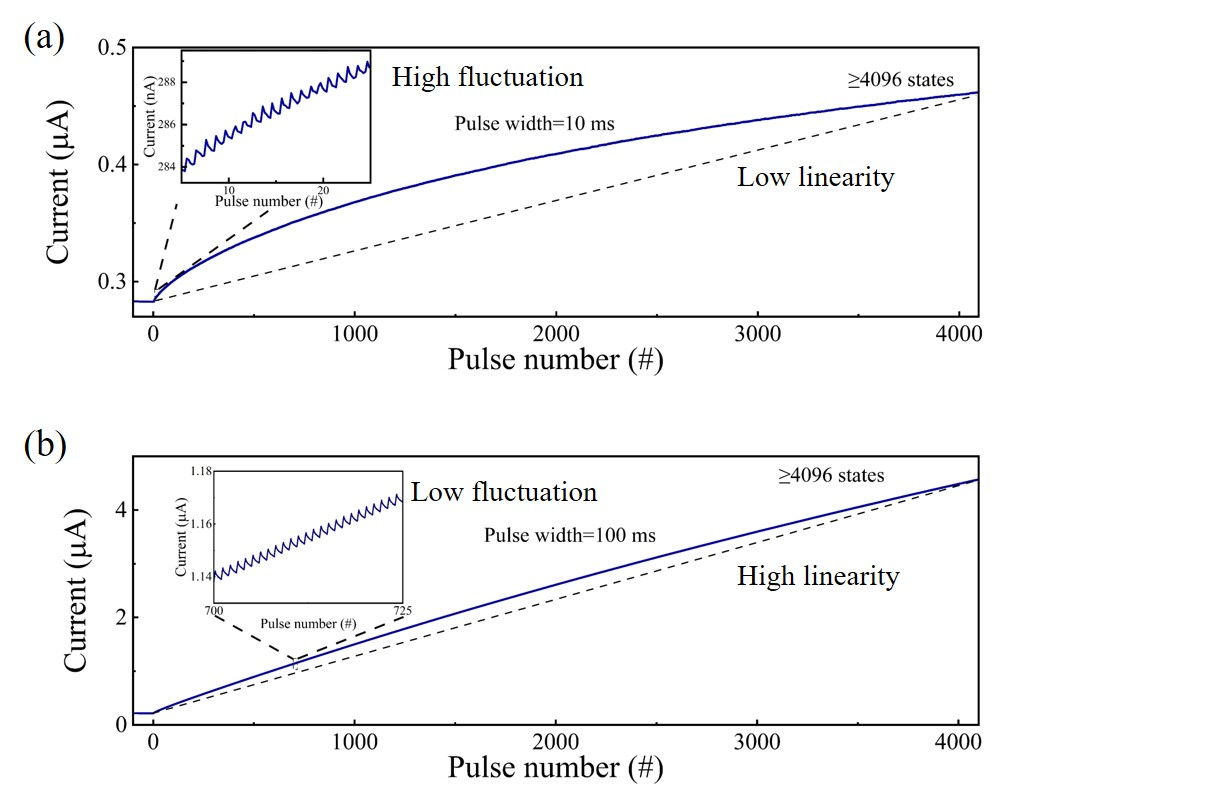


Figure S9. Conductance states under 4096 times of input optical pulses with (a) 10 ms width and (b) 100 ms width. Insets show the randomly enlarged graphs of response curve. Herein, 4096 states can be demonstrated when applying input optical pulses with 10 ms width and 100 ms width. However, the conductance variation under input optical pulses with 100 ms possesses high linearity and low flutucation, which are crucial for achieving high-accuracy image recognition.


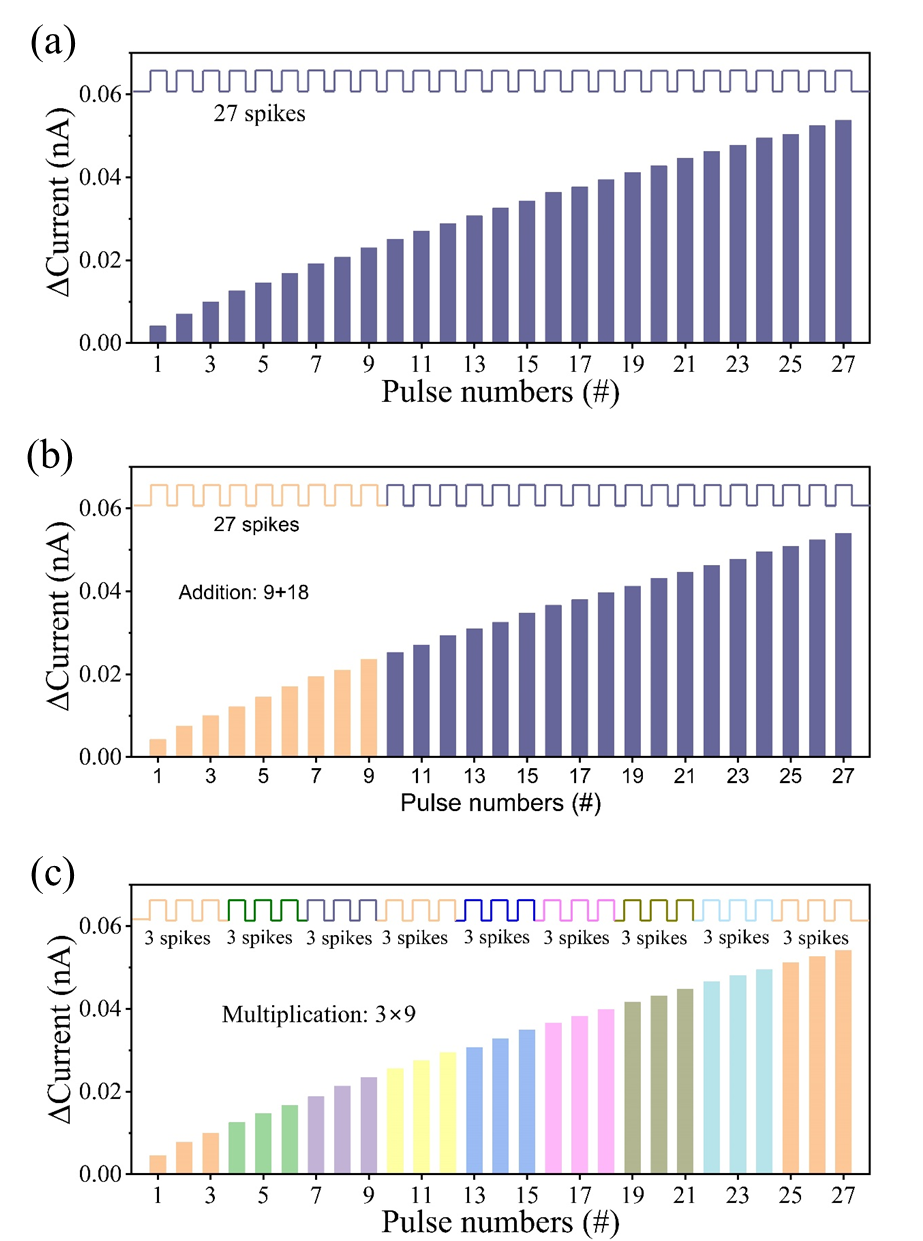


Figure S10. Arithmetic computing of addition and multiplicaiton. (a) Photocurrent change induced by optical stimulation sequence of 27 spikes. Arrtimetic computing of (b) addition and (c) multiplication by utilizing the example of 9+18 and 3×9.

As shown in Figure S10a, the photocurrent chage increases linearly as the DUV spikes increase, which reaches 0.054 nA approximately. Figure S10b demonstrates the arithmetic computing of addition with the example of 9+18, which results in the same photocurrent change of 18+9. The above result proves the commutative low of addition, i.e., 18+9=9+18. Furthermore, the photocurrent change induced by 9 sequences of 3 consecutive spikes is also equal to the *ΔI*_27_ (Figure S10c), which is consistent with the commutative low of multiplication (3×9=9×3).


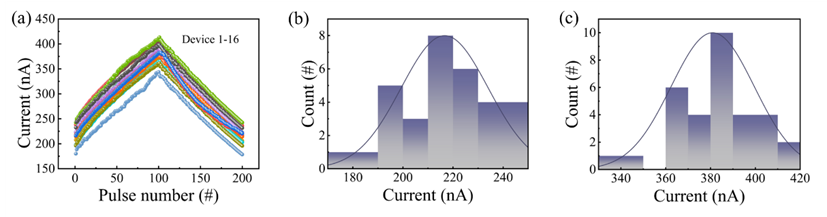


Figure S11. The reproducible properties of our Ga₂O₃-based heterojunction device. (a) The optical potentiation/electrical depression behaviors of total 16 devices were tested. (b, c) Statistical distribution of I_max_ and I_min_ of 16 devices, with fitting by a Gaussian function.

Figure S11 depicts the statitical distribution of I_max_ and I_min_ over 16 cycles, which is fitted by a Gaussian function. In order to analysis the device-to-device variability, the standard deciations (σ)/mean value (μ) are determined to be 8.03% (I_min_) and 4.87% (I_max_). All the values are below 10%, demonstrating high uniformity and stability.


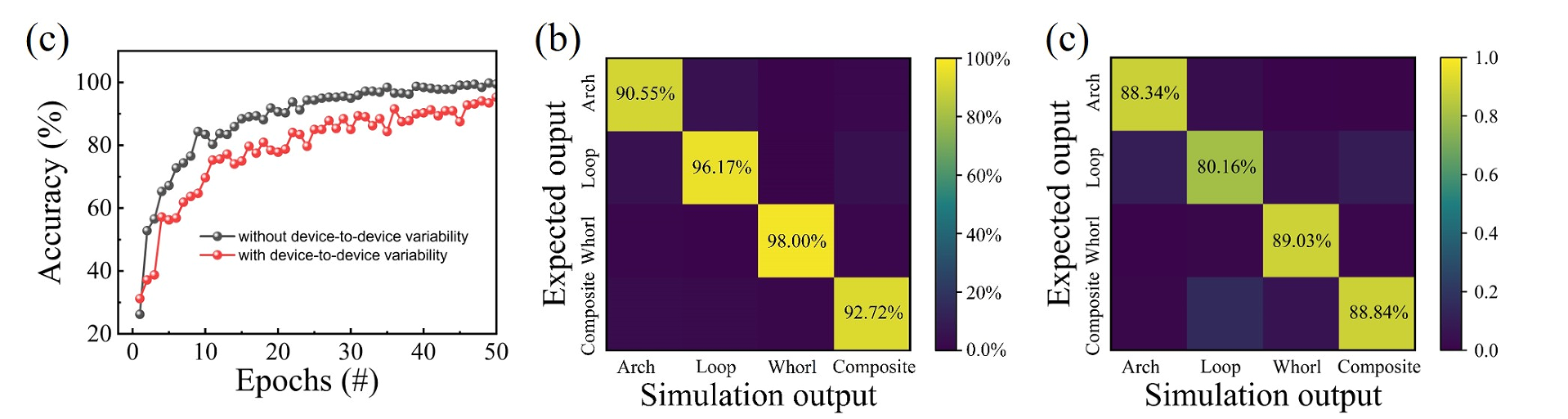


Figure S12. (a) Recognition accuracy evolution with training epochs for the NVS with the synaptic weight following one long-term potentiation and depression curve and randomly following one of 16 long-term potentiation and depression curves from 16 devices. The classification output results depicted in the confusion matrixes for the NVS (b) with the synaptic weight following one long-term potentiation and depression curve and (c) randomly following one of 16 long-term potentiation and depression curves from 16 devices.


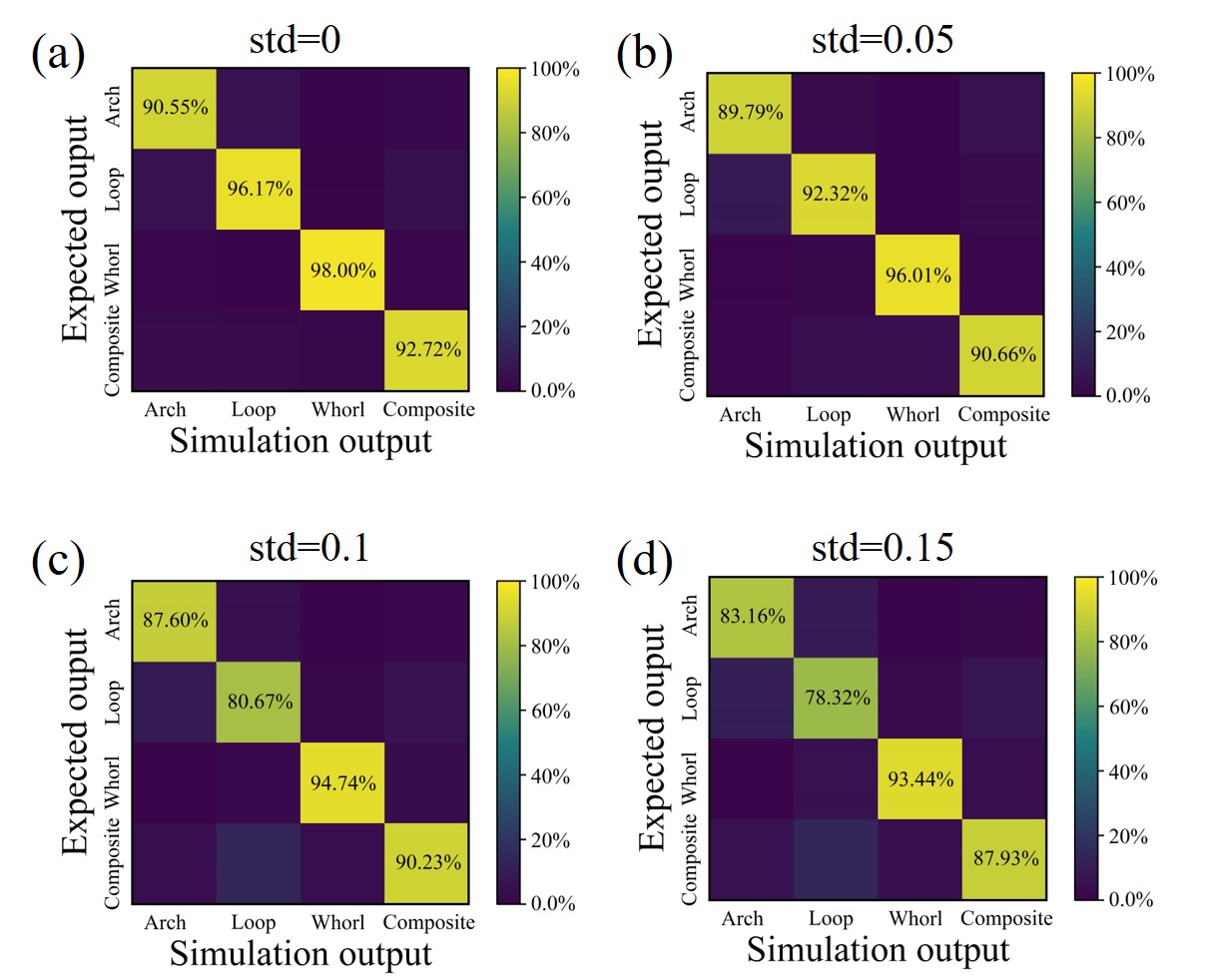


Figure S13. (a−d) Confusion matrix of the fingerprint images recognition with Gaussian noise. The mean of Gaussian random noise was set to 0, and the standard deviations (std) ranges from 0 to 0.15. The gaussian noise influences the accuracies of classification task. For the whorl pattern, the classification accuracy decreases from 98% to 93.4%, as the std increases from 0 to 0.15.

Supplementary Table S1│Comparison of dark current (I_dark_), photocurrent (I_light_), responsivity (R), and external quantum efficiency (EQE) for Ga_2_O_3_, GTO, GTO/Au/HfO_x_, and GTO/Al/HfO_x_ devices.

| Materials | I_dark_ (A) | I_light_ (A) | I_light_/I_dark_ | R (A/W) | EQE(%) |
| --- | --- | --- | --- | --- | --- |
| Ga_2_O_3_/Al | 6.5 × 10^−14^ | 3.2 × 10^−11^ | 5.0 × 10^2^ | 5.1 × 10^−5^ | 2.0 × 10^−2^ |
| GTO/Al | 1.4 × 10^−10^ | 5.9 × 10^−5^ | 4.4 × 10^5^ | 9.4 × 10^1^ | 4.6 × 10^4^ |
| GTO/Au/HfO_x_ | 2.4 × 10^−9^ | 1.2 × 10^−3^ | 5.1 × 10^5^ | 1.9 × 10^3^ | 9.3 × 10^5^ |
| GTO/Al/HfO_x_ | 1.6 × 10^−8^ | 9.4 × 10^−5^ | 6.1 × 10^5^ | 6.5 × 10^4^ | 7.3 × 10^6^ |

Supplementary Table S2│Comparison in external quantum efficiency (EQE), detectivity (D*), and responsivity of the landmark Ga_2_O_3_ devices reported in the recent literature.

| Materials | EQE (%) | D* (Jones) | R (A/W) | Ref. (#) |
| --- | --- | --- | --- | --- |
| Ga_2_O_3_ phototransistor | 1.9 × 10^6^ | 2.5 × 10^13^ | 4100 | S1 |
| In_2_O_3_/Ga_2_O_3_ phototransistor | 4.2 × 10^5^ | 1.0 × 10^16^ | 879 | S2 |
| Ga_2_O_3_ phototransistor | 2.6 x 10^6^ | 1.9 × 10^15^ | 5670 | S3 |
| Avalanche Ga_2_O_3_/ZnO | 6.0 × 10^5^ | 9.9 × 10^14^ | 1300 | S4 |
| Photoconductive Ga_2_O_3_ | 3.5 × 10^5^ | 3.9 ×10^16^ | 750 | S5 |
| Photoconductive Ga_2_O_3_ | 3.1 × 10^4^ | 8.0 × 10^15^ | 67 | S6 |
| Photoconductive Ga_2_O_3_ | 8.8 × 10^1^ | 1.0 ×10^12^ | 0.19 | S7 |
| Ga_2_O_3_ | 1.2 × 10^5^ | 3.4 × 10^14^ | 251 | S8 |
| Ga_2_O_3_ | 9.1 ×10^4^ | 1.3 ×10^16^ | 187 | S9 |
| Graphene/Ga_2_O_3_ | 1.4 × 10^4^ | 1.0 ×10^12^ | 29.8 | S10 |
| Photoconductive Ga_2_O_3_ | 1.1 × 10^5^ | 1.2 × 10^15^ | 230 | S11 |
| Photoconductive Ga_2_O_3_ | 4.4 × 10^3^ | 3.3 × 10^13^ | 8.9 | S12 |
| Graphene Ga_2_O_3_ | 1.7 × 10^4^ | 5.9 × 10^13^ | 39 | S13 |
| Photoconductive Ga_2_O_3_ | 3.2 × 10^4^ | 1.3 × 10^14^ | 70 | S14 |
| metal-Ga_2_O_3_-metal | 700 | 5.0 × 10^12^ | 1.5 | S15 |
| graphene/Ga_2_O_3_ | 23.3 | 1.9 × 10^10^ | 0.05 | S16 |
| Photovoltatic PEDOT/Ga_2_O_3_ | 139 | 1.2 × 10^12^ | 0.3 | S17 |
| Photovoltatic NTSO/Ga_2_O_3_ | 2.1 × 10^4^ | 5.0 × 10^12^ | 43 | S18 |
| MoTe2/Ta:Ga_2_O_3_ | 1.8 × 10^5^ | 3.1 × 10^12^ | 359 | S19 |
| Ga_2_O_3_/GaN | 2.6 × 10^4^ | 7.5 × 10^15^ | 56 | S20 |
| HfZrO_2_/Ga_2_O_3_ | 24 | 5.7 × 10^12^ | 0.05 | S21 |
| AFZO/Ga_2_O_3_ | 2 | 1.2× 10^11^ | 0.004 | S22 |
| LiNO_3_/Ga_2_O_3_ | 7.4 ×10^7^ | 2.7 × 10^16^ | 1.65 × 10^4^ | S23 |
| Ni/Ga_2_O_3_ | 9.34 ×10^6^ | 1.0 × 10^16^ | 1.95 × 10^4^ | S24 |
| GTO/Al/HfO_x_ | 3.0 × 10^7^ | 2.91 × 10^16^ | 6.53 × 10^4^ | This work |

**Supplementary References**

[S1] Y. Qin, S. B. Long, Q. M. He, H. Dong, G. Z. Jian, Y. Zhang, Xi. H. Hou, P. J. Tan, Z. F. Zhang, Y. J. Lu, C. X. Shan, J. L. Wang, W. D. Hu, H. B. Lv, Q. Liu, M. Liu, *Adv. Electron. Mater*. 2019, *5*, 1900389.

[S2] P. Li, J. G. Ma, H. F. Wang, W. Z. Liu, Z. S. Ju, B. S. Li, H. Y. Xu, Y. C. Liu, *IEEE Electron Device Lett.* 2023, *3*, 432.

[S3] Z. Y. Han, H. L. Liang, W. X. Huo, X. S. Zhu, X. L. Du, Z. X. Mei, *Adv. Opt. Mater.* 2020, *8*, 1901833.

[S4] B. Zhao, F. Wang, H. Y. Chen, Y. P. Wang, M. M. Jiang, X. S. Fang, D. X. Zhao, *Nano Lett.* 2015, *15*, 3988.

[S5] Y. Qin, L. H. Li, Z. A. Yu, F. H. Wu, D. A. Dong, W. Guo, Z. F. Zhang, J. H. Yuan, K. H. Xue, X. S. Miao, S. B. Long, *Adv. Sci.* 2021, *8*, 2101106.

[S6] X. H. Hou, X. L. Zhao, Y. Zhang, Z. F. Zhang, Y. Liu, Y. Qin, P. J. Tan, C. Chen, S. J. Yu, M. F. Ding, G. W. Xu, Q. Hu, S. B. Long, *Adv. Mater*. 2022, *34*, 2106923.

[S7] S. J. Cui, Z. X. Mei, Y. H. Zhang, H. L. Liang, X. L. Du, *Adv. Opt. Mater*. 2017, *5*, 1700454.

[S8] K. Arora, D. P. Singh, P. Fischer, M. Kumar, *Adv. Opt. Mater*. 2020, *8*, 2000212.

[S9] Y. F. Wang, Z. H. Lin, J. L. Ma, Y. Y. Wu, H. D. Yuan, D. S. Cui, M. Y. Kang, X. Guo, J. Su, J. S. Miao, Z. F. Shi, T. Li, J. C. Zhang, Y. Hao, J. J. Chang, *InfoMat* 2024, *6*, e12503.

[S10] S. Oh, C. K. Kim, J. Kim, *ACS Photon*. 2018, *5*, 1123.

[S11] Y. Qin, L. H. Li, X. L. Zhao, G. S. Tompa, H. Dong, G. Z. Jian, Q. M. He, P. J. Tan, X. H. Hou, Z. F. Zhang, S. J. Yu, H. D. Sun, G. W. Xu, X. S. Miao, K. H. Xue, S. B. Long, M. Liu, *ACS Photon.* 2020, *7*, 812.

[S12] Y. C. Chen, Y. J. Lu, M. Y. Liao, Y. Z. Tian, Q. Liu, C. J. Gao, X. Yang, C. X. Shan, *Adv. Funct. Mater.* 2019, *29*, 1906040.

[S13] W. Y. Kong, G. A. Wu, K. Y. Wang, T. F. Zhang, Y. F. Zou, D. D. Wang, L. B. Luo, *Adv. Mater.* 2016, *28*, 10725.

[S14] L. X. Qian, Z. H. Wu, Y. Y. Zhang, P. T. Lai, X. Z. Liu, Y. R. Li, *ACS Photon*. 2017, *4*, 2203.

[S15] A. S. Pratiyush, S. Krishnamoorthy, S. V. Solanke, Z. B. Xia, R. Muralidharan, S. Rajan, D. N. Nath, *Appl. Phys. Lett*. 2017, *110*, 221107.

[S16] Y. C. Chen, X. Yang, C. Y. Zhang, G. H. He, X. X. Chen, Q. Qiao, J. H. Zang, W. J. Dou, P. X. Sun, Y. Deng, L. Dong, C. X. Shan, *Nano Lett.* 2022, *22*, 4888.

[S17] C. Lu, M. C. Li, L. Gao, Q. H. Zhang, M. T. Zhu, X. Y. Lyu, Y. Q. Wang, J. Liu, P. Y. Liu, L. Wang, H. Y. Tao, J. Y. Song, A. L. Ji, P. G. Li, L. Gu, Z. X. Cao, N. P. Lu, *ACS Nano* 2024, *18*, 5374.

[S18] D. Y. Guo, H. Liu, P. G. Li, Z. P. Wu, S. L. Wang, C. Cui, C. R. Li, W. H. Tang, *ACS Appl. Mater. Interfaces* 2017, *9*, 1619.

[S19] G. Zeng, M. R. Zhang, Y. C. Chen, X. X. Li, D. B. Chen, C. Y. Shi, X. F. Zhao, N. Chen, T. Y. Wang, D. W. Zhang, H. L. Lu, *Mater. Today Phys.* 2023, *33*, 101042.

[S20] Y. R. Han, Y. F. Wang, S. H. Fu, J. G. Ma, H. Y. Xu, B. S. Li, Y. C. Liu, *Small* 2023, *19*, 2206664.

[S21] S. Yan, G. C. Yang, H. F. He, Q. Liu, Q. Q. Peng, J. Chen, M. K. Li, Y. M. Lu, Y. B. He, *ACS Appl. Mater. Interfaces* 2023, *15*, 22263.

[S22] P. Li, F. C. Li, J. N. Ma, D. Lin, J. G. Ma, L. Z. Ding, J. J. Guo, X. Z. Cao, J. W. Shi, H. Y. Xu, Y. C. Liu, *InfoMat* 2024, DOI: 10.1002/inf2.12607.

[S23] Q. Y. Zhang, D. M. Dong, T. Zhang, T. H. Zhou, Y. T. Yang, Y. J. Tang, J. Y. Shen, T. J. Wang, T. Y. Bian, F. Zhang, W. Luo, Y. Zhang, Z. P. Wu, *ACS Nano* 2023, *17*, 24033.

[S24] X. H. Hou, Y. Liu, S. Y. Bai, S. J. Yu, H. Huang, K. Yang, C. Li, Z. X. Peng, X. L. Zhao, X. Z. Zhou, G. W. Xu, S. B. Long, *Adv. Mater*. 2024, *36*, 2314249.
